# Water Radical Cations in the Gas Phase: Methods and Mechanisms of Formation, Structure and Chemical Properties

**DOI:** 10.3390/molecules25153490

**Published:** 2020-07-31

**Authors:** Dongbo Mi, Konstantin Chingin

**Affiliations:** Jiangxi Key Laboratory for Mass Spectrometry and Instrumentation, East China University of Technology, Nanchang 330013, China; wjmdb@hotmail.com

**Keywords:** water radical cations, water radiolysis, ab initio dynamics, DFT calculations, ultrafast chemistry

## Abstract

Water radical cations, (H_2_O)_n_^+•^, are of great research interest in both fundamental and applied sciences. Fundamental studies of water radical reactions are important to better understand the mechanisms of natural processes, such as proton transfer in aqueous solutions, the formation of hydrogen bonds and DNA damage, as well as for the discovery of new gas-phase reactions and products. In applied science, the interest in water radicals is prompted by their potential in radiobiology and as a source of primary ions for selective and sensitive chemical ionization. However, in contrast to protonated water clusters, (H_2_O)_n_H^+^, which are relatively easy to generate and isolate in experiments, the generation and isolation of radical water clusters, (H_2_O)_n_^+•^, is tremendously difficult due to their ultra-high reactivity. This review focuses on the current knowledge and unknowns regarding (H_2_O)_n_^+•^ species, including the methods and mechanisms of their formation, structure and chemical properties.

## 1. Introduction

Water is crucial for our existence on this planet and is involved in almost all biological and chemical processes [1]. The ionization of liquid water by photons, fast electrons, X-rays, heavy ions, etc., is increasingly employed in diverse fields such as photon science, radiotherapy, nuclear reactors, radiation chemistry, nuclear waste management, and so on [2,3,4,5,6,7,8,9,10].

Following the discovery of X-rays and natural radioactive phenomena, the chemistry of water radiolysis has been extensively studied for more than a century [11,12,13]. The interaction of highly energetic photons or charged particles with water results, in general, in the ejection of a quasi-free electron from the valence shell, leaving behind a positively charged radical cation, H_2_O^+•^, which then becomes stabilized as a cluster, (H_2_O)_n_^+•^. Gas-phase water radical cations can also be produced in air plasma under atmospheric pressure. Experimental data suggest that the proton transfer dynamics occur on a similar timescale as electron autoionization, with the proton transfer forming a Zundel-type intermediate [HO*…H…H_2_O]^+•^, which further ionizes to form a so-far undetected type of dicationic charge-separated species with high internal energy [14].

Characterization of generated water radical cations is usually done using mass spectrometry, which can be combined with optical spectroscopy [15,16]. Two series of cluster ions ((H_2_O)_n_H^+^ and (H_2_O)_n_^+•^) in water ice have been detected simultaneously in experiments involving secondary ion mass spectrometry (SIMS), with Au^+^, Au_3_^+^, and C_60_+ as primary ions [17]. Typically, protonated (H_2_O)_n_H^+^ cations have been observed as the predominant products of water ionization [18,19,20]. Liu et al. recently employed a microfluidic chip combined with time-of-flight secondary ion mass spectrometry (ToF-SIMS) using keV-energy ion irradiation of Bi_3_^+^ as primary ions and only detected the protonated water and heavy water clusters [21]. The observation of radical (H_2_O)_n_^+•^ cations is highly uncommon.

H_2_O^+•^ is estimated to form within a timescale of attoseconds (10^−18^ s) or subfemtoseconds at the most, based on the uncertainty relationship ΔEΔt ≈ h [22]. In pure water, the hot electrons generated after the ionization of the water relax into solvent molecules and become trapped as hydrated electron (e_hyd_^−^) species, while H_2_O^+•^ rapidly forms oxidizing •OH radicals via proton transfer, as shown in the following equation [23], which is fully accomplished in less than 1 ps.

H_2_O^+•^ + H_2_O → •OH + H_3_O^+^ (1)

In the past few decades, owing to the advent of femtosecond laser technology, several attempts using various methods have been made to identify experimentally this H_2_O^+•^ cationic species [23,24,25,26]. Nevertheless, a direct measurement of the H_2_O^+•^ decay has not yet been successfully done in pure water. It is of great interest to provide insight into the electronic signature of this radical cation by using a more sophisticated time-resolved experimental or theoretical method.

Recently, Mizuse reported the infrared spectra of water cluster radical cations (H_2_O)_n_^+•^ (n = 3–11) in the gas phase to understand the structural evolution of ionized water networks at the molecular level [15]. Further investigation has led us to have a deeper understanding of the precise structure of gaseous water radical cations (H_2_O)_n_^+•^. However, as has been pointed out, theoretical calculations often suffer from symmetry breaking, spin contamination and/or self-interaction errors in such open-shell doublet systems [27,28].

The reactivity of water exposed to ionizing radiation would be expected to depend on H-bond network structures around the created •OH radical, which often reacts with a substance via one-electron oxidation, addition and H abstraction. However, direct investigation of (H_2_O)_n_^+•^ chemistry is still extremely challenging. Except for some dynamic simulations, only a few works on the oxidation of small molecules, such as O_2_, C_2_H_4_ or CH_2_O by H_2_O^+•^ [29,30], and on other proton transfer and charge migration processes of H_2_O^+•^, have been reported.

The great interest in water radical cations and their solvated clusters has prompted a large number of studies which have greatly enhanced our understanding of this highly unstable transient state of water over recent years. This review focuses on recent studies aimed at uncovering the mechanism of formation, structural characterization and chemical properties of the water radical cation and its clusters.

## 2. Methods and Mechanisms of Formation of (H_2_O)_n_^+•^

### 2.1. Electron Bombardment

The mechanism of the generation of H^+^(H_2_O)_n_ was first proposed by Good et al. in 1970 and was based on the results of experiments involving the electron bombardment of various species [18].

First, pure nitrogen was considered. The reaction for the formation of N_3_^+^ from N^+^ has been studied very little. The N_3_^+^ bond dissociation energy has been shown to be much greater than that of N_4_^+•^ when investigated under the same conditions [31]. Good et al. found the sum of the ions N_2_^+•^ and N_4_^+•^ to constitute roughly 85% of the total ion intensity at all times during the experiments, and an almost complete conversion of N_2_^+•^ to N_4_^+•^ at long reaction times (t > 100 μs) was observed in the pressure range 0.5–4.5 torr [18].

**Figure 1:**
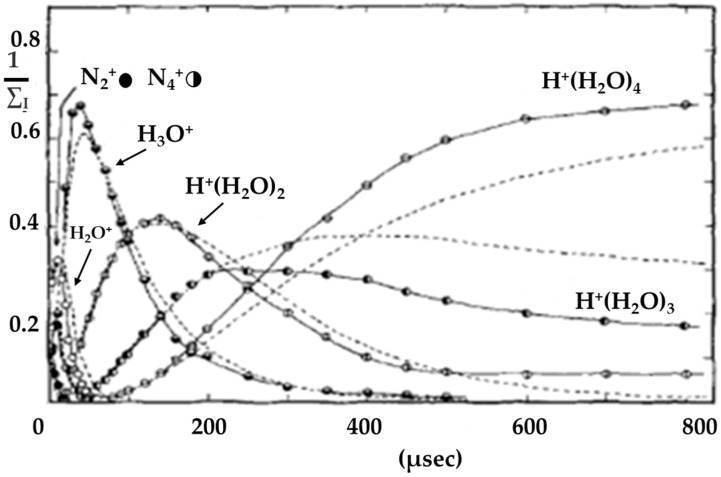
Normalized ion-intensity curves for ions in moist nitrogen after being subjected to a 10 μs electron pulse. Dashed lines represent theoretical curves calculated from integrated rate equations for consecutive reactions including reversible steps using average rate constants. Figure adapted from [18] with permission. Copyright 1970 American Institute of Physics.

Later, a series of experiments was conducted at 300 K with nitrogen containing traces of water. As shown in Figure 1, the major ions observed were N_2_^+•^, N_4_^+•^, H_2_O^+•^, H_3_O^+^, H^+^(H_2_O)_2_, H^+^(H_2_O)_3_ and H^+^(H_2_O)_4_. These ions clearly form a first-order reaction sequence. These experiments indicated a rapid decay of N_2_^+•^ while N_4_^+•^ is forming, followed by N_4_^+•^ reaching a maximum and then decaying itself, and then H_2_O^+•^ forming, reaching a maximum and falling off, etc. The final ions H^+^(H_2_O)_2_, H^+^(H_2_O)_3_ and H^+^(H_2_O)_4_ apparently reached constant concentrations after some 500–600 μs of reaction time. The authors argued that the formation of H_2_O^+•^ here could not have proceeded by a direct charge transfer from N_2_^+•^, since the disappearance of N_2_^+•^ was independent of the H_2_O concentration ([H_2_O] < 8 × 10^−20^ [N_2_]^2^) and, due to the rate constant, determined to be 1.9 × 10^−9^ cc^2^ molecule^−2^·s^−1^, being a normal value for a charge-transfer-type reaction.

H_2_O^+•^ was clearly indicated from these experiments to be the precursor of the H^+^(H_2_O)_n_ hydrates. Similarly, the rate constant of the reaction forming H_3_O^+^ from H_2_O^+•^ and H_2_O was determined to be 1.8 × 10^−9^ cc^2^·molecule^−2^·s^−1^, very close to a value of 1.6 × 10^−9^ cc^2^ molecule^−2^·s^−1^ that had been previously determined by Thynne et al. and Gupta et al. [32,33].

Perhaps most importantly, Good et al. suggested that an electron could transfer from H_2_O to N_4_^+•^ and result in the production of the primary water radical H_2_O^+•^ and then form H^+^(H_2_O)_n_ according to the following equations [18]:

N_2_ + e → N_2_^+•^ (2)

N_2_^+•^ + 2N_2_ → N_4_^+•^ + N_2_ (3)

N_4_^+•^+ H_2_O → H_2_O^+•^ + 2N_2_ (4)

**Figure 2:**
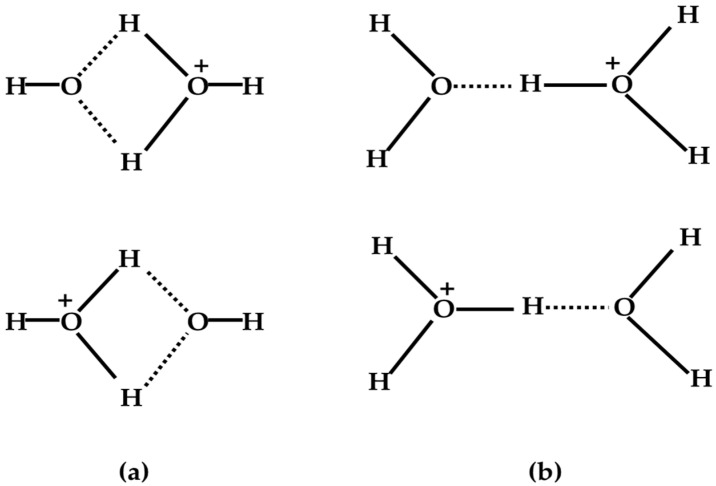
Proposed resonance structures for the (**a**) (H_4_O_2_)^+•^ and (**b**) (H_5_O_2_)^+^ ions. Figure adapted from [19] with permission. Copyright 1970 American Institute of Physics.

In the case of moist oxygen and air, in addition to the reaction mechanism mentioned above, P. Kebarle and colleagues proposed a proper alternative path by which H^+^(H_2_O)_n_ could be generated following the formation of ions with a mass of 36 amu [19], for which the notation H_3_O^+^•^•^OH was suggested by Fehsenfeld and Ferguson [34]. These ions with a mass of 36 amu were of very low intensity in all runs and could be detected only under conditions of steady electron irradiation, indicating these ions to be reaction intermediates and to play a rather central role in the reaction mechanism. Furthermore, P. Kebarle and colleagues proposed a symmetrical hybrid structure indicated by the resonance structures, as shown in Figure 2, with this structure no longer showing any distinction between the H_3_O^+^ and ^•^OH parts of the complex.

### 2.2. Corona Discharge at Atmospheric Pressure

**Figure 3:**
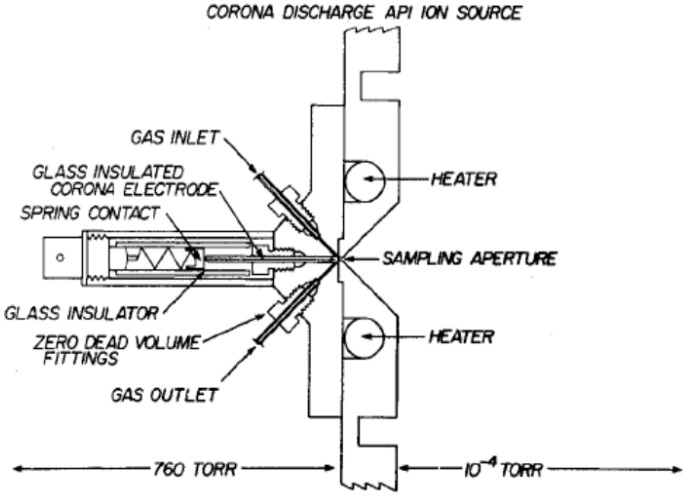
Schematic diagram of a corona discharge source with an adjustable distance between the corona discharge point and sampling aperture. Figure adapted from [35] with permission. Copyright 1976 American Chemical Society.

**Figure 4:**
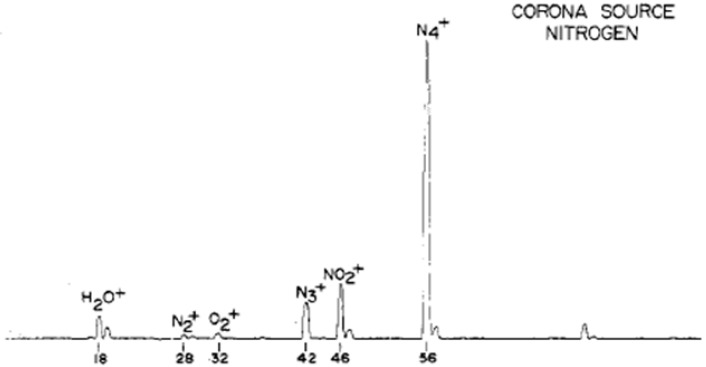
Mass spectrum of nitrogen obtained with a corona discharge point located at a distance of 0.5 mm from the sampling aperture. Figure adapted from [35] with permission. Copyright 1976 American Chemical Society.

Traces of H_2_O^+•^ were detected using a corona discharge source of the design shown in Figure 3 and with a distance of 0.5 mm from the corona discharge point to the sampling aperture. Here, the sampling procedure removed ions from the source chamber in about 10^−5^ s or less and the ions present in the source chamber under this condition of operation are shown in Figure 4 [35]. In this experiment, the ion intensity of H_2_O^+•^ was extremely low and other water radicals, i.e., (H_2_O)_n_^+•^, were not observed.

Kamabara et al. identified many clusters produced in an ambient pressure ionization (API) process through a collisional dissociation method [36]. In their study, nitrogen including trace water (including several ppm of water) was employed as the sample gas. The nitrogen flow rate was 1 L/min. A corona discharge from a needle electrode was employed for ionization and produced a total discharge current of 1 μA. The needle electrode was placed 4 mm in front of the first aperture electrode and a discharge voltage of 2.8 kV was supplied between these electrodes.

**Figure 5:**
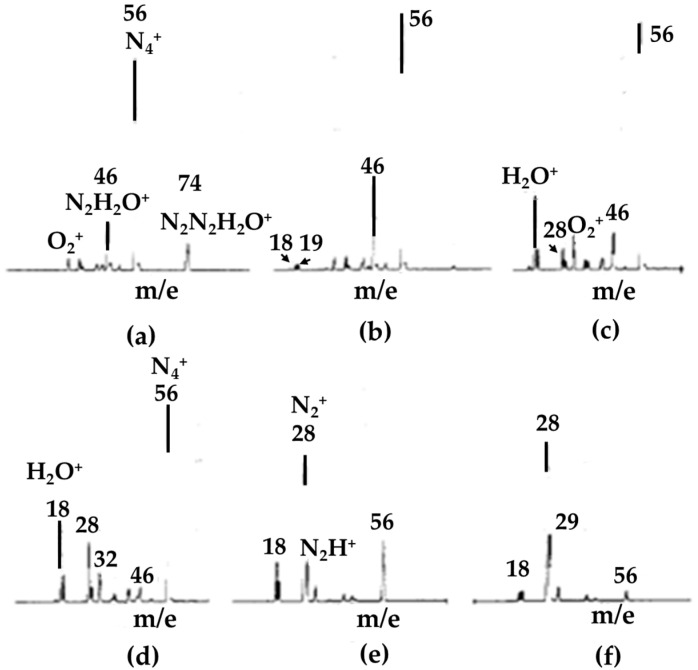
Drift voltage dependence of mass spectra for dry nitrogen. (**a**) 5 V, (**b**) 10 V, (**c**) 18 V, (**d**) 22 V, (**e**) 30 V, (**f**) 40 V. Figure adapted from [36] with permission. Copyright 1979 American Chemical Society.

Ions with masses of 74, 56 and 46 amu were observed as the major ions in the dry nitrogen streams (with less than 1 ppm water). These ions as well as N_3_^+^ have been reported in API experiments with a β-ray ionizer [37]. The types of ions present in the dry nitrogen stream were clarified by acquiring spectra over a wide range of drift voltages, as shown in Figure 5. The ions observed were identified to be N_4_^+•^ for the 56 amu peak, H_2_O^+•^ (not NH_4_^+^) for the 18 amu peak, H_2_O^+•^•N_2_ (not NO_2_^+•^) for the 46 amu peak and H_2_O^+•^•N_2_•N_2_ for the 74 amu peak. The dissociation pattern also suggested that an ion corresponding to the 74 amu peak had the form H_2_O^+•^•N_2_•N_2_ and not N_4_^+•^•H_2_O. In the API spectra for the moist nitrogen (including several ppm of water), 32, 36, 37, 42, 46, 47, 50, 55, 60, 72 and 74 amu peaks were the major ones observed.

Water clusters H^+^(H_2_O)_n_ have been reported to be the major components in this concentration range, consistent with those previously investigated using a β-ray ion source [38]. This difference was due to the complete difference between the ion resident times and effective temperature in the corona discharge ion source and those in the β-ray ion source. The resident times of ions in corona discharge ion sources have been estimated to be only tens of microseconds. Therefore, the ion–molecule reactions, such as clusters forming, cannot reach an equilibrium determined by partial pressures of the components. In addition to this, the strong electric field in the ion source would raise the effective temperature of the ions. These issues apparently caused the differences between the ionization efficiency and mass patterns of the two ion sources.

The 36 amu peak has been suggested to be probably due to two ions, one being H_3_O^+^•^•^OH and the other NH_4_^+^•H_2_O. These two species apparently dissociated in two different ways at the high drift voltages, with the clusters of H_3_O^+^•^•^OH dissociating to H_3_O^+^ and •OH (giving 19 amu ions) and clusters of NH_4_^+^•H_2_O dissociating to NH_4_^+^ and H_2_O (giving 18 amu ions). These dissociation results suggested an ion structure of H_3_O^+^•^•^OH for this species, in good agreement with previous investigations mentioned above [19,34,39].

### 2.3. Photoionization of a Water Vapor Beam for the Formation of (H_2_O)_n_^+•^ (n ≤ 3)

**Figure 6:**
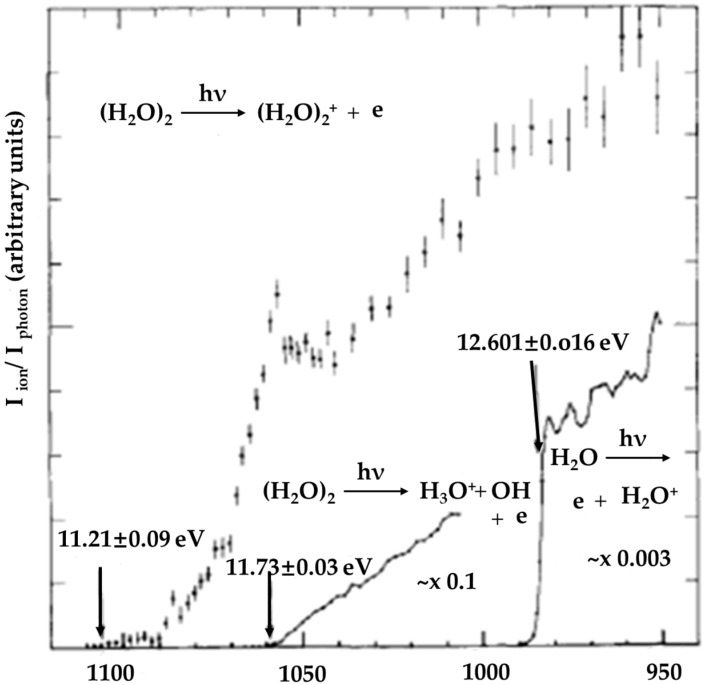
Photoion yield curves of (H_2_O)_2_^+•^, H_3_O^+^ and H_2_O^+•^ in the spectral range 95–1120 Å. Figure adapted from [40] with permission. Copyright 1977 American Institute of Physics.

The detection of (H_2_O)_2_^+•^ was reported first by Ng et al. [40], who measured the appearance potentials of (H_2_O)_2_^+•^ and its dissociated product H_3_O^+^ using molecular beam photoionization mass spectrometry. The H_2_O molecular beam was produced by seeding water vapor at 89 °C at a pressure of 150 torr of Ar and then having it expand through a 0.15-mm-diameter Pyrex nozzle. Photoion yield curves of (H_2_O)_2_^+•^, H_2_O^+•^ and H_3_O^+^ in the spectral range 950–1100 Å were acquired with a photo bandwidth of 2.5 Å full width at half maximum (FWHM) and are shown in Figure 6.

Due to the weak signal of (H_2_O)_2_^+•^, no distinct threshold was observed. The ionization potential of 11.21 ± 0.09 eV corresponded to the point where the signal fell below 0.1 count/s and thus was only an upper bound; they found that the ionization potential of the dimer shifted 1.40 ± 0.09 eV from that of the monomer [40,41]. The efficiency of the photoionization producing the (H_2_O)_2_^+•^ ion increased gradually above the threshold (11.21 ± 0.09 eV) indicative of a change in nuclear geometry. When the internal excitation of the dimer ion was increased to 0.52 eV, the H_3_O^+^ fragment ion was observed. However, no (H_2_O)_n_^+•^ cluster ions were observed. Ng et al. argued that the inability to detect larger unprotonated ions (H_2_O)_n_^+•^ (n ≥ 3) was most probably due to changes in geometry upon ionization, so that the potential minima of (H_2_O)_n_^+•^ were far from the Franck–Condon region of the shallow potential minima of the neutral (H_2_O)_n_ clusters [40].

**Figure 7:**
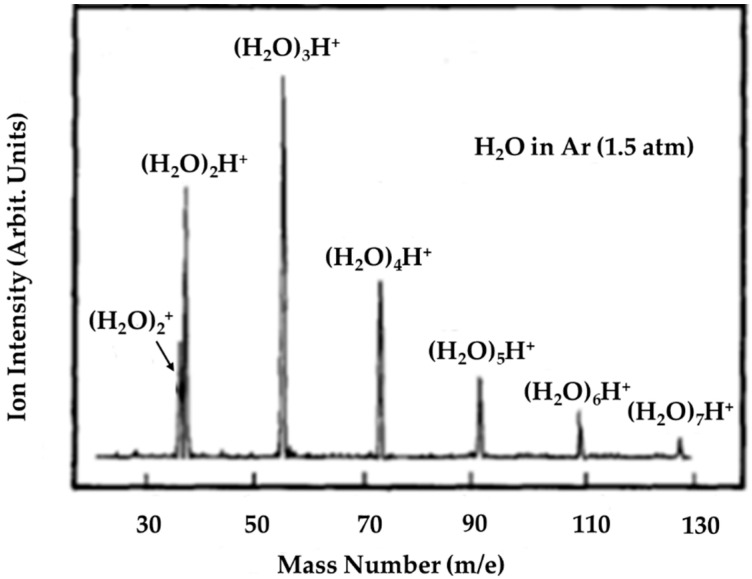
Vacuum-UV photoionization mass spectrum of water clusters at 11.83 and 11.62 eV (Ar resonance lamp; LiF window) with unit mass resolution. Conditions: 1.5 atm total stagnation pressure (seeded in Ar). Figure adapted from [10] with permission. Copyright 1986 American Institute of Physics.

Later, Shinohara et al. carried out a photoionization of supersonic cluster beams of water–argon mixtures (P ≥ 2 atm), with the vacuum-UV resonance lines at 11.83 and 11.62 eV [10]. As shown in Figure 7, with a stagnation pressure of 1.5 atm, all of the prominent peaks in the spectrum corresponded to protonated cluster ions with the general formula (H_2_O)_n_H^+^, and none corresponded to (H_2_O)_2_^+•^.

**Figure 8:**
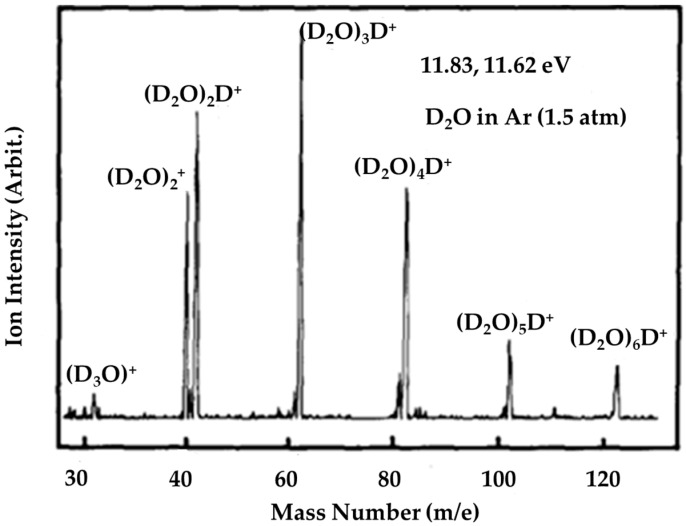
Ar lamp (LiF window; 11.83 and 11.62 eV) photoionization mass spectrum of heavy water clusters (D_2_O)n with unit mass resolution. Conditions: 1.5 atm total stagnation pressure (seeded in Ar). Figure adapted from [10] with permission. Copyright 1986 American Institute of Physics.

Deuterium substitution studies were carried out by Shinohara et al. [10]. As shown in Figure 8, the change in isotope had no effect on the ratio (D_2_O)_2_^+•^/(D_2_O)_2_D^+^, and Shinohara et al. argued that proton transfers within water clusters were not affected by the deuterium substitution [10].

Tunable vacuum ultraviolet (VUV) photoionization studies of water clusters were performed using 10–14 eV synchrotron radiation and analyzed using reflectron time-of-flight (TOF) mass spectrometry, devised by Belau et al. [42]. Three series of peaks comprising unprotonated and protonated water clusters and their metastable fragments were distinguished in the spectrum. However, only two unprotonated water species were indicated here, namely H_2_O^+•^ and (H_2_O)_2_^+•^, and they were observed at extremely low ion intensity.

In all of the experiments reported so far, the protonated cluster ions were by far the predominant ions indicated by the mass spectra, whereas the unprotonated cluster ions were either not detectable or yielded peaks that were orders of magnitude weaker than those corresponding to protonated ions. According to an acquired water dimer photoelectron spectrum [43], the 11.83 eV photon can effectively produce the first vertical ionization, which has been interpreted as the ejection of an electron from the out-of-plane nonbonding molecular orbital (MO) (2a’’) localized on the proton donor oxygen atom. Tomoda and Kimura have suggested the potential minimum for (H_2_O)_2_^+•^ to be far from the wide Franck–Condon region expected from the shallow potential minimum of (H_2_O)_2_ [44]. They have also suggested the presence of some reaction paths with no activation energy barriers between the vertically ionized points [(H_2_O)_2_^+•^]^*^_vip_ and the dissociation limiting point yielding H_3_O^+^ and ^•^OH.

### 2.4. Photoionization of a Molecular Beam for the Formation of (H_2_O)_n_^+•^ (n > 3)

**Figure 9:**
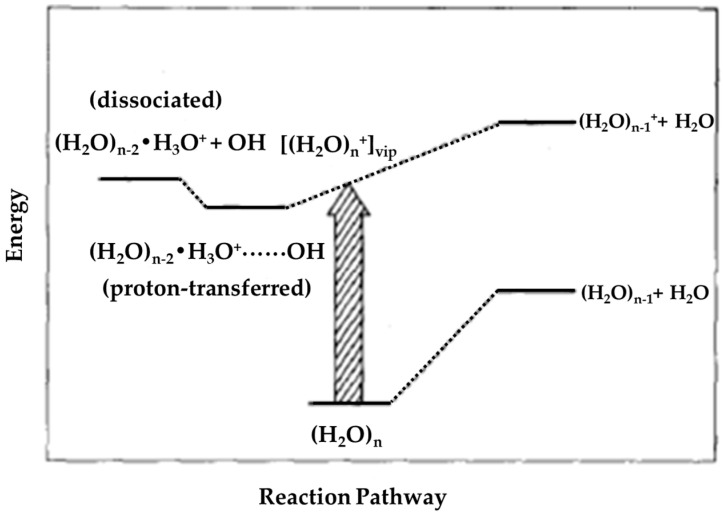
Schematic energy level diagram of water clusters (H_2_O)_n_ along the reaction channels. The label “vip” stands for vertically ionized points (sic). Figure adapted from [10] with permission. Copyright 1986 American Institute of Physics.

Shinohara et al. [10] expected the failure to observe (H_2_O)_n_^+•^ to imply the occurrence of geometrical changes upon ionization. The geometries of (H_2_O)_n_^+•^ species differ quite considerably from those of (H_2_O)_n_ due to the shorter O-O distances of the ionized form and thus the Franck–Condon transitions from the potential curves of the neutral (H_2_O)_n_ clusters do not cover the potential minima of (H_2_O)_n_^+•^ along the proton transfer coordinates [44,45]. They concluded that in water clusters, except for (H_2_O)_2_^+•^, the Franck–Condon excitations from the neutral clusters (H_2_O)_n_ to the corresponding ionic states cannot produce the parent cluster ions (H_2_O)_n_^+•^, even in the near-threshold ionization, as illustrated in Figure 9.

**Figure 10:**
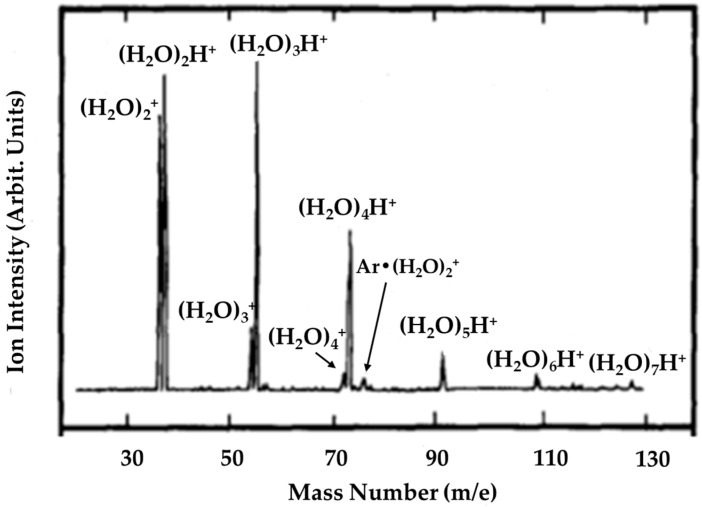
Vacuum-UV photoionization mass spectrum of water clusters at 11.83 and 11.62 eV (Ar resonance lamp; LiF window) with unit mass resolution. Condition: 3.0 atm stagnation pressure and 200 accumulations. Figure adapted from [10] with permission. Copyright 1986 American Institute of Physics.

However, (H_2_O)_n_^+•^ (2 ≤ n ≤ 10) and (Ar)_m_•(H_2_O)_n_^+•^ (2 ≤ m ≤ 3; 2 ≤ n ≤ 7) were detected in mass spectra, exhibited in Figure 10, for the first time when supersonic cluster beams of water–argon mixtures were photoionized, with the vacuum-UV resonance lines in the near threshold at higher stagnation pressures (≥3 atm). Further investigation demonstrated that, at a much higher stagnation pressure of 5 atm, the quantity of unprotonated water clusters increased markedly, to such an extent that the signal intensities of (H_2_O)_n_^+•^ exceeded those of the corresponding protonated cluster ions (H_2_O)_n_H^+^. Shinohara et al. [10] also compared the mass spectra of the supersonic H_2_O beam seeded in Ar by electron impact with those subjected to 11.83 eV photons, but in the latter case, (H_2_O)_n_^+•^ was not observed, suggesting a lack of production of unprotonated ions at high energies.

Finally, based on systematic studies, Shinohara et al. [10] concluded that carrying out near-threshold photoionization with Ar as a “cooling gas” is necessary for the generation of the (H_2_O)_n_^+•^ ions and they proposed a formation mechanism involving (H_2_O)_n_^+•^ species being generated via the photoionization of water–argon binary clusters, (Ar)_m_•(H_2_O)_n_ + hγ → (H_2_O)_n_^+•^ + mAr + e^−^. Here, the excess energies produced during ionization can be randomized within the [(Ar)_m_•(H_2_O)_n_^+•^]^*^_vip_ clusters (so-called intra-cluster excess energy dissipation) and finally be used to release argon atoms, giving rise to the stable (H_2_O)_n_^+•^ ions and various (Ar)_k_•(H_2_O)_n_^+•^ ions produced by the near-threshold photoionization, suggesting the effective Franck–Condon excitations of the neutral (Ar)_m_•(H_2_O)_n_ clusters to the related [(Ar)_m_•(H_2_O)_n_^+•^]^*^_vip_. They further claimed that (H_2_O)_n_^+•^ ions could also be observed via ionization of the (CO_2_)_m_•(H_2_O)_n_ [46] binary system as well as (N_2_O)_m_•(H_2_O)_n_ clusters.

Shiromaru et al. [47] determined the appearance potentials for (H_2_O)_2_^+•^, (H_2_O)_3_^+•^, (H_2_O)_2_H^+^ and (H_2_O)_3_H^+^ using synchrotron radiation. The results suggested that water clusters in their experiment were directly ionized since the photoionization efficiency curve of H_2_O^+•^ showed no distinct autoionization structure. Furthermore, in contrast to the (H_2_O)_2_^+•^ ion, (H_2_O)_3_^+•^ was not observed to have been generated from the water trimer, a result attributed to the Franck–Condon region covering a small part of the potential minimum for the water trimer ion as compared to that for the dimer ion. As for the threshold ionization produced using synchrotron radiation, the Franck–Condon assumption having restricted direct vertical ionization involving the ladder climbing mechanism was in agreement with the results obtained by Shinohara et al. [10] to rationalize the lack of unprotonated water clusters.

**Figure 11:**
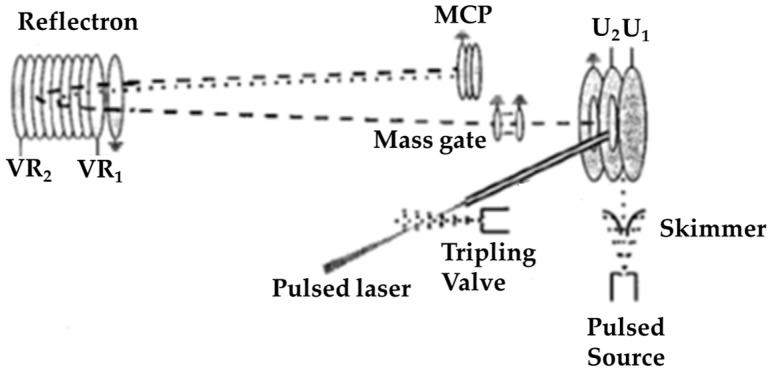
Schematic overview of the molecular beam photoionization reflectron TOF mass spectrometer used by Jongma et al. Figure adapted from [48] with permission. Copyright 1998 American Chemical Society.

Hydrogen-bonded water clusters were formed with inert gases adsorbed to them in a strong molecular beam expansion carried out by Jongma et al. [48]. A schematic overview of the molecular beam photoionization reflectron TOF mass spectrometer that they used is shown in Figure 11. A room-temperature mixture of water vapor and carrier gas (total backing pressure between 2 and 6 bar) was expanded through a pulsed (200 μs duration) solenoid valve (general valve). The standard orifice of the valve was replaced with a gradually sloped conical nozzle (smallest diameter: 0.8 mm) to enhance water cluster formation. The gas mixture was expanded into the source chamber (pumped by an 1100 L/s turbomolecular pump). Under these conditions, the cooling during the expansion allowed for the efficient production of clusters containing up to ~80 water monomers per cluster. After passing a 0.5 mm diameter skimmer, the molecular beam entered the ionization region (pumped by a 400 L/s turbomolecular pump) of the reflectron TOF mass spectrometer. Single-photon ionization of the water clusters was carried out with vacuum-ultraviolet radiation. The water cluster ions were extracted by the applied electric fields into the drift tube. A mass gate mounted in this tube was used to select part of the spectrum. Parent and daughter ions were separated in time in the reflectron and detected using an microchannel plate (MCP) detector after passing a field-free region.

**Figure 12:**
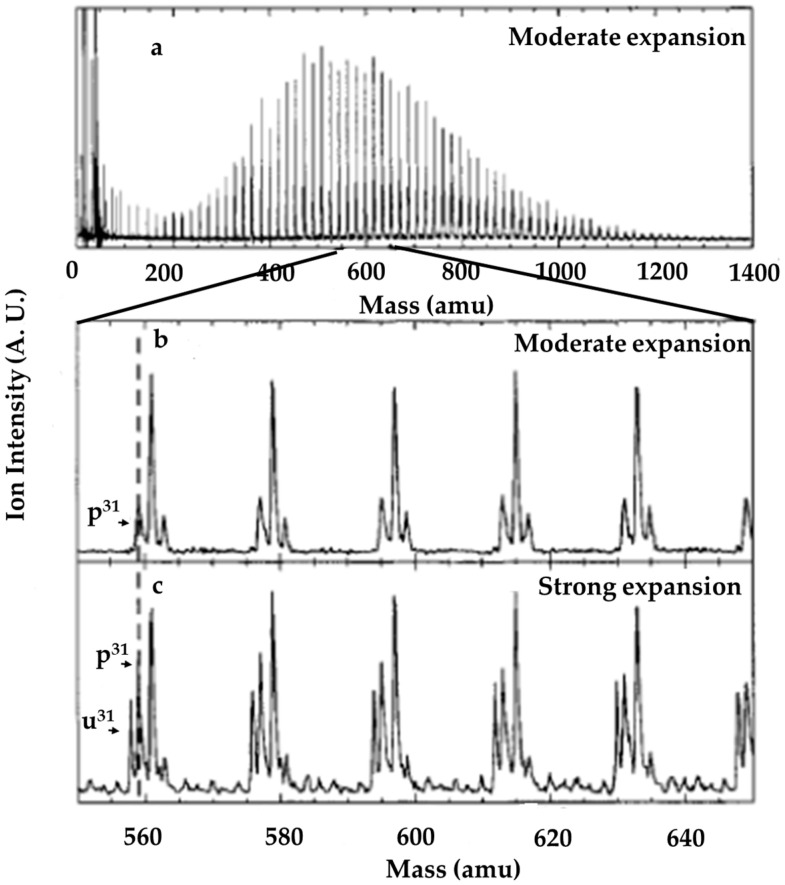
Molecular beam photoionization reflectron TOF mass spectrometer detected by Jongma et al. (**a**) Mass spectrum as recorded using a weak H_2_O/Ar expansion. (**b**) Small part of the same spectrum as displayed in (**a**), starting at p^31^. A progression of “triplets” separated by 18 amu is clearly seen. The first peak of each group is the parent p^n^ ion for n = 31–36. The second and third peaks are daughter ion peaks, produced by the loss of one and two water monomers from the protonated parent ion, respectively. (**c**) Mass spectrum recorded under strong expansion conditions. An extra peak with a mass that is 1 amu less than that of the p^n^ parent is observed and assigned as the “unprotonated” water cluster. The small peaks between the quartets are the same unprotonated ions with varying numbers of Ar atoms attached to them. Figure adapted from [48] with permission. Copyright 1998 American Chemical Society.

Figure 12a shows a typical mass spectrum obtained by Jongma et al. [48], as recorded under modest expansion conditions using Ar as a carrier gas (backing pressure: 2 bar). Cluster ions, each containing up to about 75 water molecules were readily observed. Evidence of in source decay (ISD), as an asymmetric peak shape with a tail to longer flight times (higher masses when converted to the mass scale), was clearly observed, as shown in Figure 12b [48]. Asymmetric peak shapes were clearly observed for the protonated water clusters (abbreviated as P^n^ in the figure, such as P^31^). Furthermore, a mass spectrum composed of a series of strong quartets with additional weak mass features between the quartets was obtained after the backing pressure and driving voltage of the pulsed source were increased, as shown in Figure 12c. The low-mass members of the quartets appeared 1 amu lower than the P^n^ family. This family of mass features nominally has the formula (H_2_O)_n_^+•^ and is formally designated as U^n^ (shown in the figure, such as U^31^).

**Figure 13:**
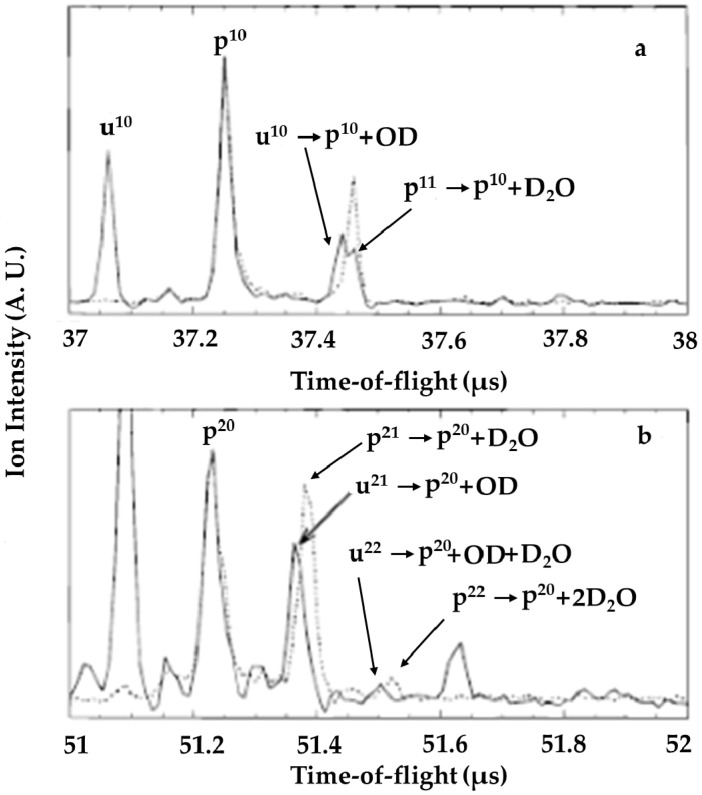
Molecular beam photoionization reflectron TOF mass spectrometer detected by Jongma et al. (**a**) Small part of the TOF spectrum obtained using a D_2_O/N_2_ expansion. Spectra with (solid line) and without (dashed line) unprotonated cluster signals present are superimposed and scaled to the intensity of the p^10^ peak. The double peak at 37.45 μs (solid line) is due to loss of OD from the unprotonated cluster (u^10^) and due to the loss of D_2_O from the protonated cluster (p^11^). This assignment is confirmed by the dotted spectrum, where only p^11^ → p^10^ + D_2_O is possible. (**b**) Similar to (**a**) for larger clusters. The presence of the peak due to loss of D_2_O from the protonated cluster is hardly detectable under conditions where the unprotonated species is present. The small peak to the right of this (solid line) is due to the daughter ion produced by the loss of OD followed by the loss of D_2_O from the unprotonated cluster. The peak position of daughter ions due to the loss of two D_2_O monomers from protonated clusters is at a slightly different position, as is obvious from the dashed spectrum, and is not observed in spectra where the unprotonated clusters appear (solid line). Figure adapted from [48] with permission. Copyright 1998 American Chemical Society.

Unambiguous evidence for H_3_O^+^•(H_2_O)_n−1_•^•^OH as the structure of U^n^ was provided by the observation of a loss of •OH from U^n^, as shown in a TOF spectrum between 37 and 38 microseconds (Figure 13a) [48]. Even clearer evidence for this conclusion was provided by the TOF spectrum between 51 and 52 microseconds (Figure 13b), i.e., at around the arrival time of U^20^ and P^20^ (solid trace). Here, evidence for the occurrence of U^21^ → P^20^ + •OD was provided by inspection of the solid trace of this spectrum, with a 20 ns difference between the two peaks (the solid line peak and the dotted line peak around 51.4 μs) clearly visible. Further analysis of the heavy water cluster ions by Jongma et al. [48] revealed the loss of •OD from U^n^ to be much more probable than the loss of D_2_O. The domination of the metastable decay by •OD loss was consistent with an •OD molecule having been formed in the proton transfer reaction and then having diffused away from the charge center of the cluster, as discussed in detail below.

Jongma et al. [48] also confirmed that the formation of unprotonated ions would be possible only during expansion conditions strong enough to produce neutral water clusters with adsorbed Ar. To more fully explore this phenomenon, the authors acquired photoionization mass spectra for several carrier gases, including Kr, O_2_, N_2_, CO_2_ and even CO. All of these carrier gases exhibited behaviors qualitatively similar to that of Ar. In all cases, the U^n^ family was always detected simultaneously with U^n^M_m_ families (M = neutral carrier gas molecules, Kr, O_2_, N_2_, CO_2_ and even CO). This result was consistent with the idea that mixed neutral clusters must first form in the molecular beam expansion before unprotonated water clusters can be observed. However, Jongma et al. [48] disagreed with the point of view regarding the “active parent ion cooling mechanism” proposed by Shinohara et al., [10] at least for the large clusters. Jongma et al. attributed this phenomenon to rapid evaporative cooling. Once the energy in the cluster ion becomes low enough, the mobility of the •OH radical in the cluster would be sufficiently reduced so that the •OH radical would become trapped in the cluster, leading to the formation of “unprotonated” clusters. Such a conclusion could actually be proven under favorable conditions in a post source decay (PSD) experiment.

Cluster ion distributions of water in a molecular beam were investigated by Radi et al. using femtosecond ionization at 780 nm and reflectron time-of-flight mass spectrometry since vertical ionization from the ground state to the potential energy surface of the [(H_2_O)_n_^+•^]^*^ ion could be achieved [49]. Furthermore, since intramolecular energy transfer in intermediate states is not expected during ionization, cluster heating in femtosecond photoionization is likely to be insignificant. However, unprotonated species of the form (H_2_O)_n_^+•^ have not been observed, with the exception of the dimer ion (H_2_O)_2_^+•^, which was attributed by Radiet al. to the Franck–Condon-restricted direct vertical ionization.

**Figure 14:**
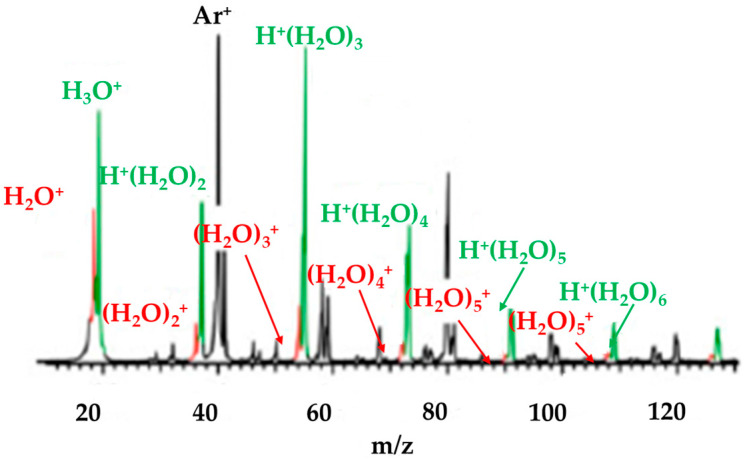
Mass spectrum of ions produced using the present ion source. Figure adapted from [15] with permission. Copyright 2011 The Royal Society of Chemistry.

Mizuse et al. [50] reported IR spectra of (H_2_O)_n_^+•^ (n = 3–11) water cluster cations; specifically, they acquired photodissociation spectra by using a tandem quadrupole mass spectrometer and a coherent IR source. IR spectra of (H_2_O)_n_^+•^•Ar (n = 3–7) were also acquired using essentially the same technique. As shown in Figure 14, H^+^(H_2_O)_n_ and H^+^(H_2_O)_n_•Ar yielded much stronger peaks than did (H_2_O)_n_^+•^ and (H_2_O)_n_^+•^•Ar. In this study, the water cluster cations (H_2_O)_n_^+•^ and (H_2_O)_n_^+•^•Ar were generated in a supersonic jet expansion. A gaseous mixture of H_2_O (trace; ^17,18^O-depleted H_2_^16^O, 99.99% ^16^O, ISOTEC) and Ar (5 MPa) was expanded into a vacuum chamber through a high-pressure pulsed valve (Even–Lavie valve) [51]. The gas pulse was crossed by a 200 eV electron beam from an electron gun (Omegatron) in the collisional region of the jet. Cluster ions formed became larger and were cooled following the collisions. The cluster ion of interest was selected by the first mass spectrometer (with a mass resolution (Δm) of ~ 1) and was irradiated with coherent IR light. IR spectra were acquired by monitoring the photofragment intensity as a function of the IR wavelength. The second mass spectrometer was tuned to select the fragments of both the H_2_O-loss and the •OH-loss channels (Δm ~ 3) for spectroscopy of bare (H_2_O)_n_^+•^.

In addition to using the “colder” cluster source, Mizuse et al. further tried a “warmer” ion source, i.e., with a lower stagnation pressure that should reduce the degree of collisional cooling [50]. They argued that cooling efficiency and the choice of the carrier gas are critical to the yields of (H_2_O)_n_^+•^.

**Figure 15:**
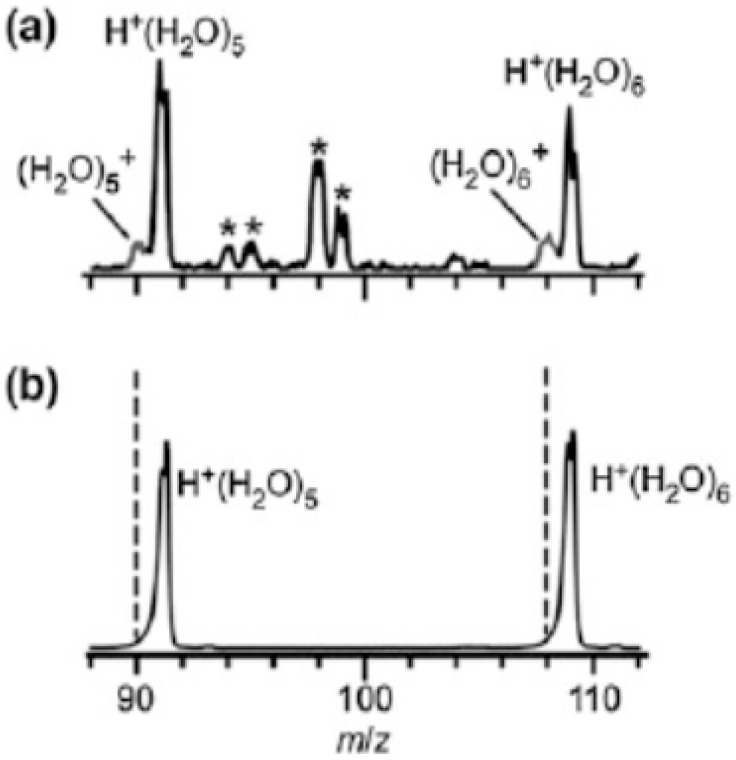
Cluster ion distributions obtained with (**a**) a “colder” cluster ion source and (**b**) “warmer” cluster ion source. Figure adapted from [15] with permission. Copyright 2011 The Royal Society of Chemistry.

Figure 15 shows a comparison of typical mass distributions resulting from using the “colder” and “warmer” ion sources. For the “warmer” ion source, only H^+^(H_2_O)_n_ was observed, consistent with the previous mass spectrometry studies by Shinohara et al. and Jongma et al. discussed above [10,48]. As for the “colder” ion source, carrier gas dependencies were also checked by Mizuse et al. [50]. When Ne was used as a carrier gas instead of Ar, the relative yields of (H_2_O)_n_^+•^ were lower. In the case of He, only a very small amount of (H_2_O)_n_^+•^ was produced, also in agreement with previous mass spectrometry studies [48].

**Figure 16:**
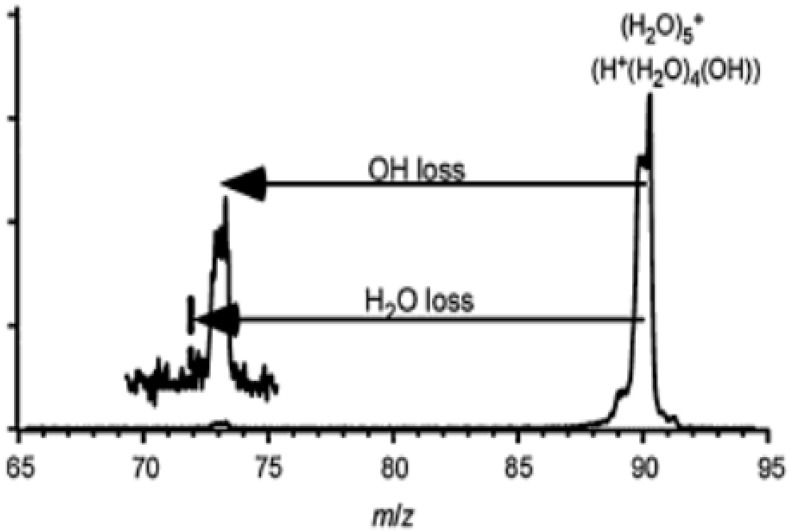
Infrared photodissociation mass spectrum of (H_2_O)_5_^+•^. Figure adapted from [15] with permission. Copyright 2011 The Royal Society of Chemistry.

Figure 16 shows an IR photodissociation mass spectrum of (H_2_O)_5_^+•^. Mizuse et al. [15] selected this ion using the first mass filter and then (H_2_O)_5_^+•^ was irradiated by an IR light pulse of 3734 cm^−1^. The second mass filter then analyzed the *m*/*z* ratios of the resulting ions, and then the spectrum was obtained. The IR light of this photon energy was resonant with the antisymmetric stretch of the terminal water moiety. With the measurement, only the •OH-loss fragment was found actually to contribute to the observed IR spectra, at least for (H_2_O)_3–5_^+•^. This result implied the presence of H^+^(H_2_O)_n−1_^•^OH-type structures.

### 2.5. Stabilization of (H_2_O)_n_^+•^ Produced by Electron Impact in Helium Nanodroplets

**Figure 17:**
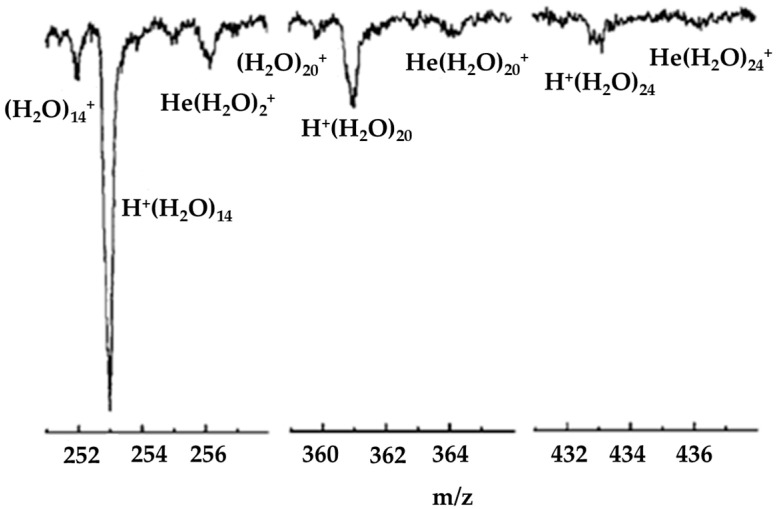
The n = 14, 20 and 24 regions of a mass spectrum recorded from water clusters (H_2_O)_n_ in helium nanodroplets. Figure adapted from [55] with permission. Copyright 2007 American Institute of Physics.

Rapidly removing excess energy is required for (H_2_O)_n_^+•^ to survive the ionization process. An alternative means of preventing the dissociation of the parent cluster ion is to carry out the ionization in a helium nanodroplet. Helium nanodroplets provide a low equilibrium temperature (0.4 K), possess an exceptionally high thermal conductivity and can dissipate energy rapidly as a result of evaporative loss of weakly bound helium atoms [52]. The release of gas phase (H_2_O)_n_^+•^ cluster ions from this environment has previously been reported by Lewerenz et al. [53] and Fröchtenicht et al. [54]. The investigation by Fröchtenicht et al. was concerned primarily with recording infrared spectra of neutral water clusters and recording electron impact mass spectra, in which only much weaker signals due to unprotonated water cluster ions were reported. The study by Lewerenz et al. also involved electron impact ionization mass spectrometry. Relatively low-resolution mass spectra were obtained, yet signals corresponding to unprotonated water cluster ions were reported as shoulders on the much stronger H^+^(H_2_O)_n_ peaks. As shown in Figure 17, Yang et al. [55] more closely investigated the electron impact mass spectrometry of water clusters in helium nanodroplets. Not only did they report seeing signals corresponding to both H^+^(H_2_O)_n_ and (H_2_O)_n_^+•^, as reported previously, but they also reported the observation of He(H_2_O)_n_^+•^ cluster ions for the first time. Based on ab initio calculations, Yang et al. suggested that, as was the case for H^+^(H_2_O)_n_ ions, the preferential location for a positive charge in large (H_2_O)_n_^+•^ clusters is at the surface of the cluster, specifically on a dangling O-H bond to which a single helium atom can attach via a charge-induced dipole interaction.

### 2.6. Formation of (H_2_O)_n_^+•^ Using High-Energy Photons or Particles

**Figure 18:**
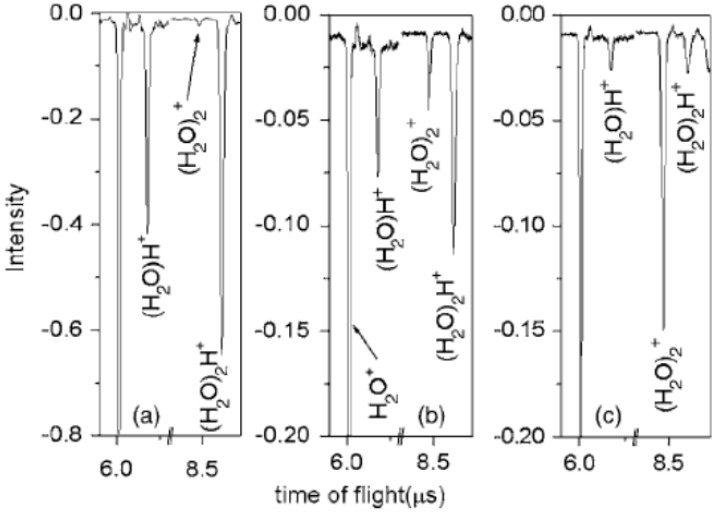
TOF mass spectra of unprotonated water dimer ion formed in different carrier gases: (**a**) pure He gas, (**b**) 5% Ar mixed in He gas and (**c**) 20% Ar mixed in He gas, respectively. Figure adapted from [56] with permission. Copyright 2006 American Institute of Physics.

A tabletop soft X-ray laser was applied for the first time by Dong et al. as a high-energy photon source for chemical dynamics experiments in the study of water [56]. Specifically, a 26.5 eV soft X-ray laser (pulse duration of ~ 1 ns) was employed. As shown in Figure 18a, when pure He was used as the carrier gas, a weak signal for (H_2_O)_2_^+•^ was observed on the low-mass side of (H_2_O)_2_H^+^, the daughter ion of (H_2_O)_3_^+•^. When 5% Ar was mixed into the He expansion gas, the (H_2_O)_2_^+•^ signal strengthened and the H_3_O^+^ signal weakened (see Figure 18b). For a 20%Ar/80% He expansion, the (H_2_O)_2_^+•^ mass feature was much larger than that for H_3_O^+^ (Figure 18c). The intensity of the signal corresponding to the protonated cluster ion (H_2_O)_2_H^+^ also decreased as the concentration of Ar in the binding gas was increased. Single photon ionization by a 26.5 eV photon has much more energy (ca. 15 eV) than that required for ionization of all the water clusters. If this energy were to remain in the clusters, only H_2_O^+•^ and its fragments could be observed. Nonetheless, (H_2_O)_2_^+•^ was observed even with no Ar present in the expansion. This observation suggests that almost all of the excess energy in these clusters (ca. 15 eV) was removed by the exiting electron, consistent with the arguments proposed by Shiromaru et al. [47].

**Figure 19:**
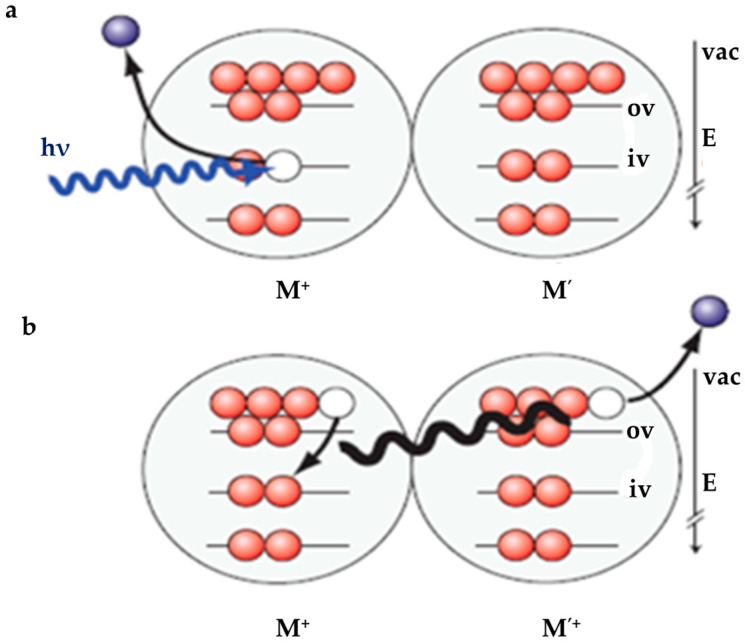
Schematic of intermolecular coulombic decay (ICD) of an inner-valence vacancy in a hydrogen-bonded network of water molecules. This interatomic autoionization process takes place when an inner-valence vacancy created by (photo)ionization on molecule M (**a**) is filled by an electron from an outer-valence orbital of the same molecule, while another outer-valence electron is emitted from the nearest-neighbor molecule M’ (see **b**). Figure adapted from [57] with permission. Copyright 2010 Springer Nature.

At even higher energies, new reaction channels would open; e.g., ionizing the inner valence electrons in water would lead to the subsequent autoionization process identified as intermolecular coulomb decay (ICD) [57,58]. The unequivocal signature of this process has been observed in simultaneous measurements of low-energy electrons and photoelectrons generated from inner-valence shells using vacuum-ultraviolet light. ICD occurs when the excited particle is only loosely attached to neighboring particles by, for example, van der Waals forces or hydrogen bonding (as shown in Figure 19).

**Figure 20:**
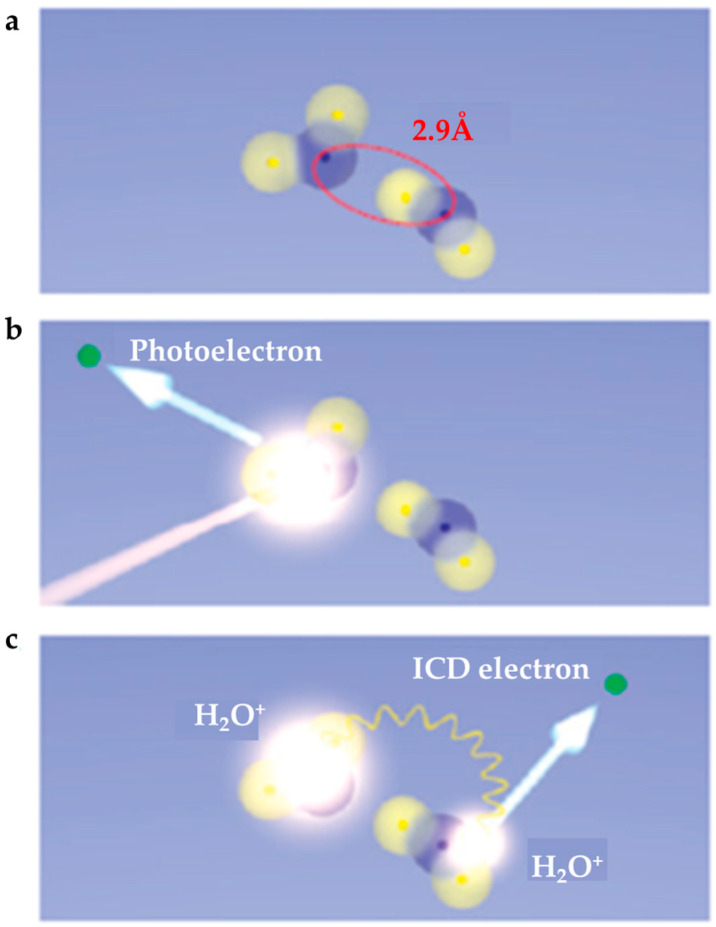
(**a**) Geometry of the water dimer. (**b**,**c**) An electron from the inner valence shell of one of the molecules of the dimer is ejected by absorption of a photon (**b**) and then the energy released by de-excitation at this site is transferred to the neighboring site, where a second, low-energy electron is emitted. Figure adapted from [58] with permission. Copyright 2010 Springer Nature.

Jahnke et al. reported the direct observation of an ultrafast transfer of energy across the hydrogen bridge in (H_2_O)_2_, illustrated in Figure 20 [58]. They argued that this decay is faster than the proton transfer that is usually a prominent pathway in the case of electronic excitation of small water clusters and leads to dissociation of the water dimer into two H_2_O^+•^ ions.

Electron impact ionization measurements with 70 eV electrons [55] have shown the formation of H_2_O^+•^ radical cations with a yield of 37.5% (with the rest indicated to be H_3_O^+^). As a result of the impacts of these high-energy electrons, the electrons from all valence orbitals were indicated to be ejected. In the experiment carried out by Jahnke et al. [58], 100% of the autoionization events resulted from the ICD process, but the efficiency of the ICD process in the water dimer was not determined. As Svoboda et al. have noted [59], it is difficult to estimate the fraction of the water dimers with the ionized 2a_1_ electrons that are deactivated via the internal conversion process without becoming further ionized. The results of the chemical calculation simulations done by Svoboda et al., together with the electron impact measurements of Buck and Winter [60], could provide a hint regarding the efficiency of the ICD process. Svoboda et al. made a rough estimate based on the assumption that all of the valence electrons would be equally likely to become ionized by a 70 eV electron. The efficiency of the ICD process was estimated in this way to be between 20% and 40%. Since fast nuclear dynamics could take place even upon 1a_1_ ionization [14,59] as well as upon 2a_1_ electron ionization, the system can then efficiently lose potential energy, thus closing the autoionization channel and hence reducing the ICD process efficiency to much lower than 100%.

## 3. Structural Properties of (H_2_O)_n_^+•^: Simulations and Experimental Studies

**Figure 21:**
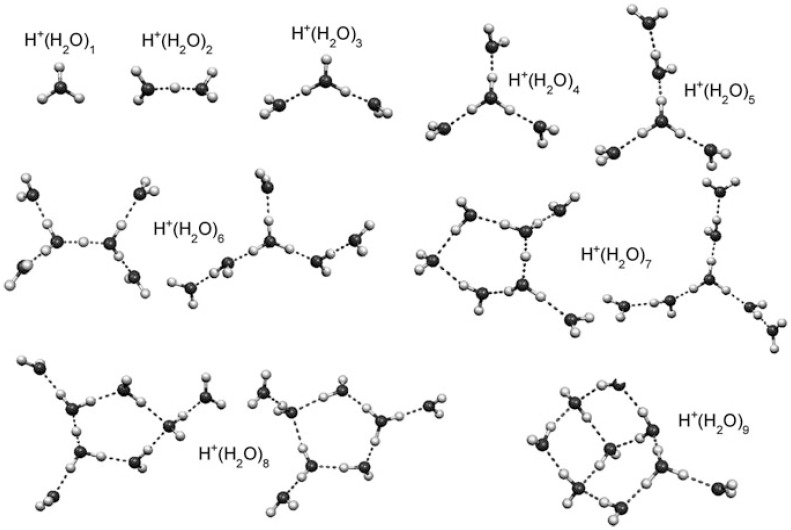
Experimentally characterized structures of H^+^(H_2_O)_n_. Figure adapted from [15] with permission. Copyright 2011 The Royal Society of Chemistry.

Studies of clusters have provided detailed insights into structural trends and dynamics of hydrogen-bonded water networks, as shown in Figure 21 [61,62,63,64,65,66,67,68,69,70,71,72].

Another advance in studying hydrated clusters in the gas phase is to understand chemical reactions in aqueous solutions from microscopic observations, since there are circumstances in which condensed phase studies may be unable to adequately investigate such detailed reaction mechanisms. Reducing the number of molecules in a cluster system can provide deeper insights into such a complicated system and has been used to characterize the reaction mechanism of an important chemical reaction: ionizing radiation-induced reactions of water [73,74,75,76,77,78,79,80,81,82,83].

Structures of (H_2_O)_n_^+•^ would be expected to reflect the inherent instability of H_2_O^+•^ and the reactivity of cationized water networks. Attaining such knowledge is quite important for understanding ionizing-radiation-induced chemistry in water. However, although (H_2_O)_n_^+•^ has been produced in trace amounts in several experimental studies, structural information on these species is still limited. This section focuses on this problem.

### 3.1. Investigation on the Structure of the Water Dimer Radical Cation (H_2_O)_2_^+•^

The structure of (H_2_O)_2_^+•^ is very important in radiation chemistry [12,84,85], as is its role in the deionization of O_2_^+•^ in the ionosphere [39,86]. Two structural motifs have been suggested. In early theoretical works, the difference between the energy of the optimized dimer cation and that of the cation with the optimal geometry of the neutral dimer calculated at the Mőller-Plesset second-order perturbation theory (MP2) level was found to be almost the same as that calculated at the Unrestricted Hartree-Fock theory (UHF) level, specifically 2.57 and 2.56 eV, respectively [87]. This result may indicate that the electron correlation is important for estimating the absolute stabilization energy of the cation, but not so much with regards to geometry optimization [88], suggesting that (H_2_O)_2_^+•^ would adopt a charge-resonance-stabilized structure reminiscent of hydrazine [H_2_O…OH_2_]^+•^, i.e., a so-called dimer cation [89].

Recent fine theoretical studies were consistent with the presence of a structure resulting from proton transfer. Gill and Radom characterized the ^2^A’’ state of the hydrogen-bonded (C_s_ symmetry) isomer and the ^2^B_u_ state of the hemi-bonded (C_2h_ symmetry) isomer as two minima at the MP4/6-311G (MC)**//MP2/6-31G* level of theory [90]. The energy difference between the isomers was found to be 8.9 kcal/mol. Sodupe, Oliva and Bertran located five structures [91] of the ionized water dimer (H_2_O)_2_**^+•^** and argued for a similarity between its Cs symmetry transition state and its C_1_ minimum structure, but with the •OH rotated out of the plane. The energy difference between these two structures was predicted to be 0.03 kcal/mol.

Cheng et al. [92] determined fourteen stationary points for the water dimer radical cation on its doublet electronic state potential energy surface by carrying out harmonic vibrational frequency analyses using coupled cluster theory with single and double excitations (CCSD) and CCSD with perturbative triple excitations (CCSD (T)). Two stationary points were found to be local minima: isomer 1 (C_1_ symmetry), with H_3_O^+^-^•^OH character (hydrogen-bonded system), and isomer 7 (C_2_ symmetry), with [H_2_O…H_2_O] ^+•^ character (dimer cation).

**Figure 22:**
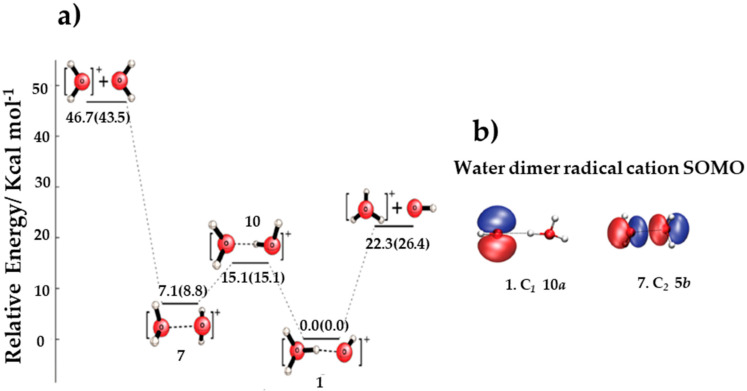
Illustrations of the proton transfer and dissociation process for the dimer cations. (**a**) Schematic of the potential energy surface (in kcal/mol, zero-point vibrational energy (ZPVE) corrected values in parentheses) showing proton transfer and dissociation processes for the dimer cation and hydrogen-bonded water dimer radical cation structures 1, 7 and 10 at the aug-cc-pVQZ CCSD (T) level of theory. (**b**) Singly occupied molecular orbitals (SOMO) of the (H_2_O)_2_^+•^ equilibria 1 and 7 [92]. Figure adapted from [92] with permission. Copyright 2009 American Chemical Society.

Adiabatic energies of the ionization of (H_2_O)_2_ to isomers 1 and 7 were determined to be 10.81 and 11.19 eV, respectively, with the former being in excellent agreement with the experimental value of 10.8–10.9 eV. The critical dissociation energy of isomer 1 to H_3_O^+^ and •OH was predicted to be 26.4 kcal/mol, while the dissociation energy of isomer 7 to H_2_O^+•^ and H_2_O was determined to be 34.7 kcal/mol. As shown in Figure 22, at the aug-cc-pVQZ CCSD (T) level of theory, the hydrogen-bonded 1 and hemi-bonded 7 minima were determined to be separated by 8.8 kcal/mol, with an interconversion barrier (1 → 10 → 7) of 15.1 kcal/mol.

For several cases, particularly the binary ion/water complexes involving OH^−^, O^−^, F^−^, or H_3_O^+^, Gardenier et al. reported binding energies considerably greater than one would expect for a typical ion/water complex with bonding dominated by ion/dipole or dispersion interactions [93,94] as well as hydrogen bonding. These large interaction energies reflect an electronic structure in which the shared proton is partially transferred from the water molecule to the molecular ion. The extent of this effect can be characterized by values of ρ, as illustrated in the following equation:

ρ = [r_XH_ − r_XH_^0^] − [r_X’H_ − r_X’H_^0^] (5)

where X represents the acceptor (or donor) molecule and X’ represents the donor (or acceptor) molecule. The r_XH_ is the XH bond length in the X-H-X’ complex, and r_XH_^0^ is the value in the isolated molecule or molecular ion, calculated at the same level of theory; this equation was first reported by Scheiner and used by Leopold and A. Johnson to characterize proton delocalization and vibration-induced proton transfer [95,96,97,98]. By this definition, larger values of |ρ| reflect greater localization of the shared proton, whereas values close to zero suggest equal sharing. A. Johnson and his co-workers quantified the extent of proton delocalization by using wave functions that were obtained in the analysis of the spectrum [99] and found this extent to be much smaller than that of the binary ion/water complexes reported earlier, indicative of the initially localized bridging proton exploring an intra-cluster proton transfer configuration to enhance the covalent nature of the H-bond to the proton acceptor and meaning that this species would be best described as an H_3_O^+^-^•^OH ion-radical complex.

**Figure 23:**
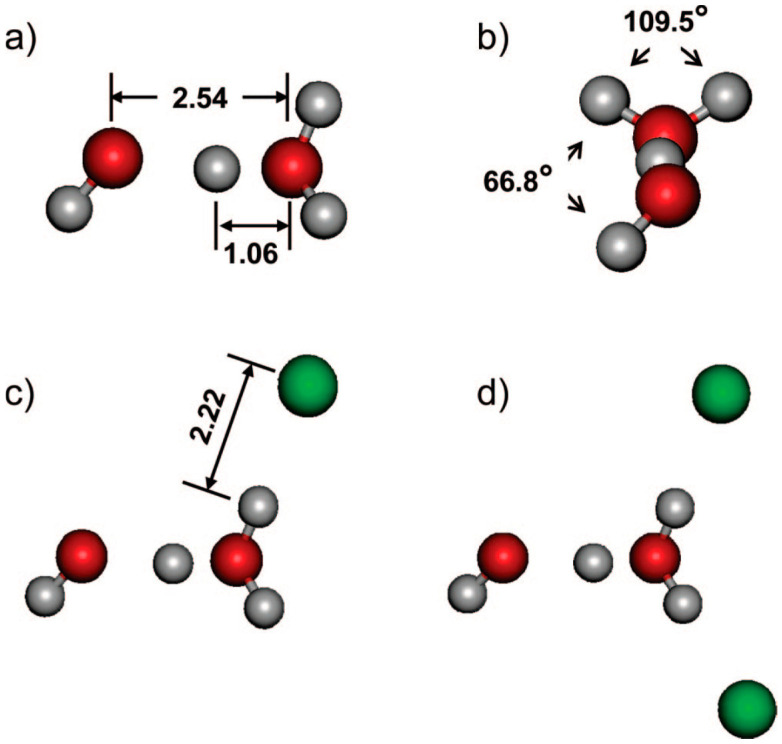
(**a**,**b**) The equilibrium structures of (H_2_O)_2_^+•^ reported by Pieniazek et al. [101]. (**c**,**d**) The structures of (H_2_O)_2_^+•^•Ar_n_ with (**c**) n = 1 and (**d**) n = 2 reported by Gardenier et al. [99]. Selected bond lengths are reported in Ångstroms. Figure adapted from [99] with permission. Copyright 2009 American Chemical Society.

**Figure 24:**
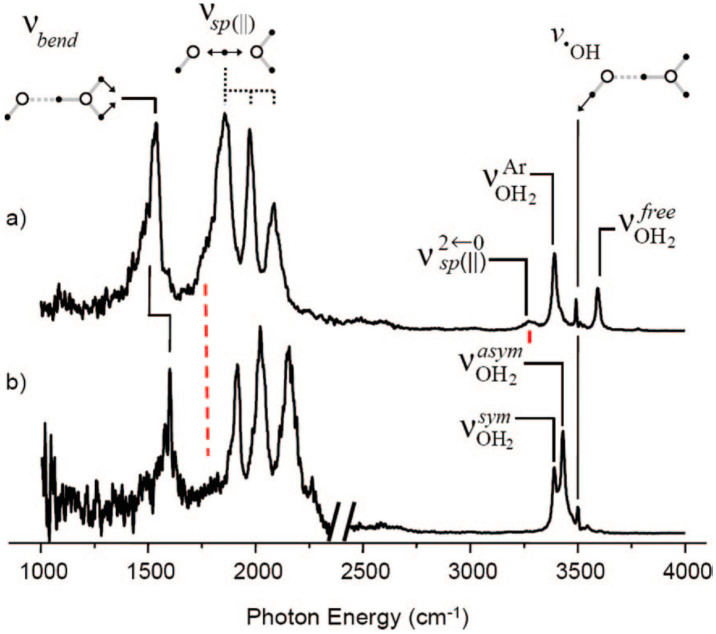
Vibrational predissociation spectra of (**a**) (H_2_O)_2_^+•^•Ar, monitoring the loss of Ar and (**b**) (H_2_O)_2_^+•^•Ar_2_, monitoring the loss of two Ar species per molecule for excitations above 2400 cm^−1^ and the loss of one Ar in the low-energy region. Schematic displacement vectors are included to illustrate qualitative assignments of observed features. The subscript sp (ll) refers to the parallel displacement of the bridging proton along the heavy-atom (O-O) axis. The superscripts sym and asym denote, respectively, the symmetric and asymmetric stretches of dangling OH groups of the H_3_O^+^ moiety, each of which is bound to an Ar atom in the n = 2 system. The subscript ^•^OH refers to the OH stretch on the hydroxyl moiety. Calculated anharmonic values for υ_sp_ (ll) and 2υ_sp_ (ll) are shown with dashed red lines. Figure adapted from [99] with permission. Copyright 2009 American Chemical Society.

Collisional studies of the (H_2_O)_2_^+•^ ion in the gas phase by Stace and co-workers [100], for example, were more consistent with an [H_3_O^+^-^•^OH]ion-radical structure than with the dimer cation structure [61,99]. This ion-radical structure (shown in Figure 23) was also indicated to be the minimum-energy arrangement in a theoretical paper by Pieniazek et al. [101]. Very recently, Gardenier et al. presented IR spectra of argon-tagged (H_2_O)_2_^+•^ and (H_2_O)_2_^+•^ and they only observed the proton-transferred [H_3_O^+^-^•^OH] type (shown in Figure 23) [99]. They found the resulting bands involving the displacement of the bridging proton to be broad and appear as a strong triplet centered around 2000 cm^−1^, with this observation completely ruling out a contribution from a hydrazine-like isomer. Other observed band positions are shown in Figure 24.

### 3.2. Structural Trends for Water Radical Cations (H_2_O)_n_^+•^ (3 ≤ n ≤ 10 and n > 10)

Though confirmation of the formation of the •OH radical in the dimer cation is an indication of important progress, the ion-core structure in larger clusters was at this stage still ambiguous because the ion-core structure often changes with solvation. Further experimental studies were thus required to elucidate the general trends of the network structures and the interplay between the protonated site and the •OH radical under the hydration environment.

Based on MP2/UHF/4-31++G** data, Novakovskaya et al. arranged the H_3_O^+^ and •OH fragments and divided them into two groups: either the •OH fragment acting exclusively as a proton acceptor in all of its hydrogen bonds or its being directly bonded to H_3_O^+^ and acting also as a proton donor in an H-bond with a water molecule [88]. They concluded the structures of the first group to be slightly more stable, with the H_3_O^+^ fragment tightly bonded to water molecules. For larger clusters, more of the positive charge would be shared by water molecules, but the charge of each molecule would decrease with increasing n and with increasing distance between the molecule and H_3_O^+^ fragment. In contrast, the H-bond between •OH and neighbor fragments would gradually weaken with increasing cluster size.

**Figure 25:**
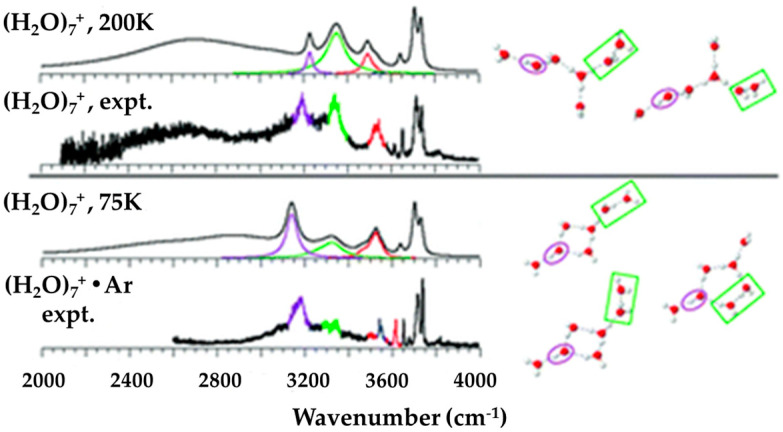
Infrared spectra of neat and Ar-tagged (H_2_O)_7_^+•^ compared with simulated spectra of (H_2_O)_7_^+•^ at 200 K and 75 K [102]. Figure adapted from [102] with permission. Copyright 2014 The Royal Society of Chemistry.

Besides IR spectra, the structure evolution of ionized water radical cations (H_2_O)_n_^+•^ with n = 5–8 was studied using ab initio methods by M.-K. Tsai, J.-L. Kuo and their co-workers. Both the size and temperature dependence of the structure of (H_2_O)_n_^+•^ and solvation of the •OH radical were analyzed systematically by using a structure searching method based on the previous understanding of the hydrogen bond (H-bond) networks in neutral and protonated water clusters (Figure 25).

**Figure 26:**
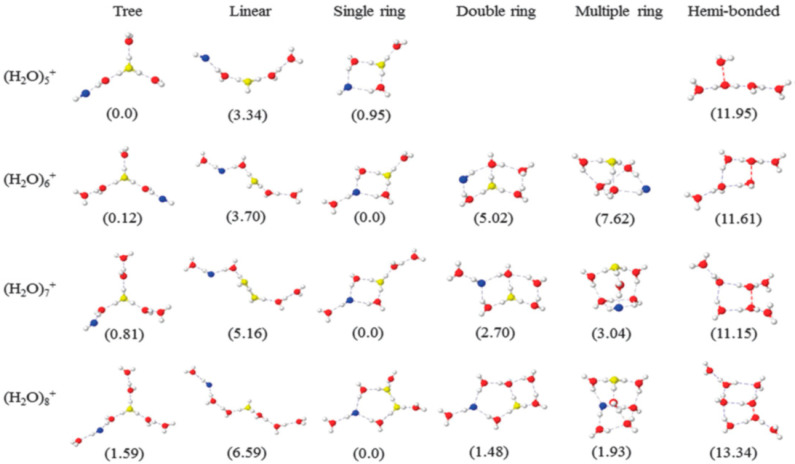
The most stable structures of (H_2_O)_n_^+•^ in different topological groups, as determined using ab initio methods. To guide the eyes, the O atoms of H_3_O^+^, the •OH radical and H_2_O are shown in yellow, blue and red. The relative energies with zero-point energy (ZPE) correction (in kcal/mol) are shown in parentheses. Figure adapted from [102] with permission. Copyright 2014 The Royal Society of Chemistry.

The agreement between the calculated and experimentally determined IR spectra in the free •OH stretch region confirmed the preference of the •OH radical to stay on the terminal site of the H-bond network for n = 5 and n = 6. Furthermore, M.-K. Tsai, J.-L. Kuo and their co-workers found that the •OH radical began to form H-bonds with water molecules as an H-bond donor for n = 7 and 8 (as shown in Figure 26).

Vibrational signatures of fully solvated •OH were found to be located at 3200–3400 cm^−1^, coinciding with the additional peaks found in previous experimental data obtained by Mizuse et al. [15].

**Figure 27:**
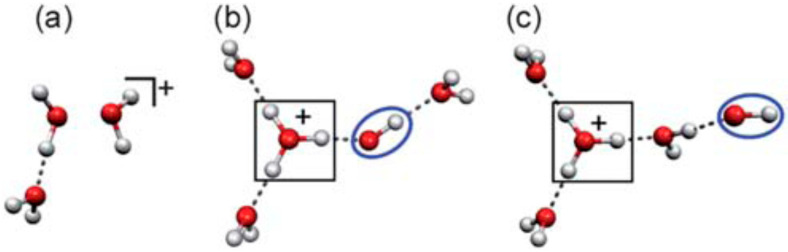
Three different structural motifs of (H_2_O)_n_^+•^ (n ≥ 3). These structures were constructed from previous results [48,100,103]. The protonated site is indicated by a border. The •OH radical moiety is circled. (**a**) “Dimer cation core”-type structure. (**b**) Structure with the H_3_O^+^ ion in contact with the ^•^OH radical. (**c**) Structure with the ion and radical separated from each other by solvent. Figure adapted from [15] with permission. Copyright 2011 The Royal Society of Chemistry.

Besides the theoretical calculation, three groups have reported structural motifs (for n ≥ 3) from their mass spectrometric analyses, but with these three motifs being different from each other [48,100,103]. Figure 27 shows the structural motifs constructed based on their results. In Figure 27, structure (a) shows a solvated “dimer cation core” type, as suggested by Yamaguchi et al. No •OH radical exists in this type [103]. Both structures (b) and (c) are of the proton-transferred type, but with these two differing in the position of the •OH radical. In structure (b), suggested by Angel and Stace, the H_3_O^+^ ion and •OH radical are in contact with each other and fully hydrated via three hydrogen bonds [100]. However, in structure (c), suggested by Jongma et al., the ion-radical pair is dissociated, with the •OH radical located at the terminus of the network [48]. Structure (a) is expected to display a reactivity very different from the reactivities of structures (b) and (c) due to the absence of the •OH radical. Furthermore, if the •OH radical is formed as in structures (b) and (c), its location relative to the protonated site may be a crucial factor for the reactivity of ionized water networks because the mobility of the •OH radical may depend on whether or not the radical is tightly bound to the protonated (charged) site.

**Figure 28:**
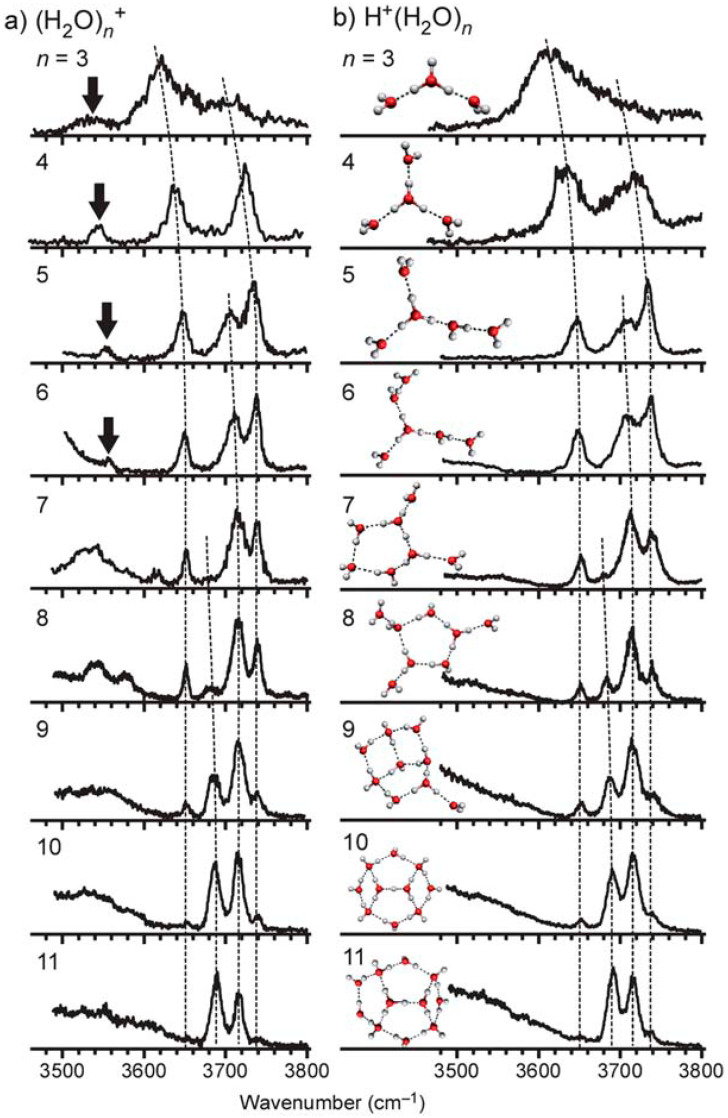
(**a**) IR spectra of (H_2_O)_n_^+•^ (n = 3–11) in the free OH stretch region. (**b**) IR spectra and representative structures of (H_2_O)_n_H^+^. (Dotted curves mark the locations in each spectrum, from the low frequency side, of a symmetric stretch of 1-coordinated water, a free OH stretch of 3-coordinated water, a free OH stretch of 2-coordinated water and an antisymmetric stretch of 1-coordinated water, respectively). Figure adapted from [15] with permission. Copyright 2011 The Royal Society of Chemistry.

To reveal the structures of (H_2_O)_n_^+•^ at the molecular level, such as network shapes, ion core motifs and the location of the •OH radical, Mizuse carried out a systematic investigation combining IR spectroscopy, especially in the OH stretch region, and the inert gas (such as Ar) attachment technique [50]. In contrast to the case for the H^+^(H_2_O)_n_ system, free OH stretch band patterns of (H_2_O)_n_^+•^ were found to be quite similar to those of protonated water clusters (H_2_O)_n_H^+^ (shown in Figure 28b) reported by several groups so far [16,69,71,104,105,106,107,108,109].

However, the spectra of (H_2_O)_n_^+•^ were noted to not be exactly identical to those of (H_2_O)_n_H^+^ in the 3500–3600 cm^−1^ region: an additional band was observed for (H_2_O)_3–6_^+•^ and it is indicated by the arrow in Figure 28. The frequency of this band was too low to be attributed to any free OH stretch of the H_2_O moieties, as the gas-phase frequency of the stretch vibration of the •OH radical has been observed to be 3570 cm^−1^. Mizuse et al. assigned this band to the free stretch of an •OH radical moiety in the clusters. This assignment was consistent with the •OH radical stretch frequencies in (H_2_O)_2_^+•^• Ar _1,2_ of 3511 and 3499 cm^−1^, respectively [99].

**Figure 29:**
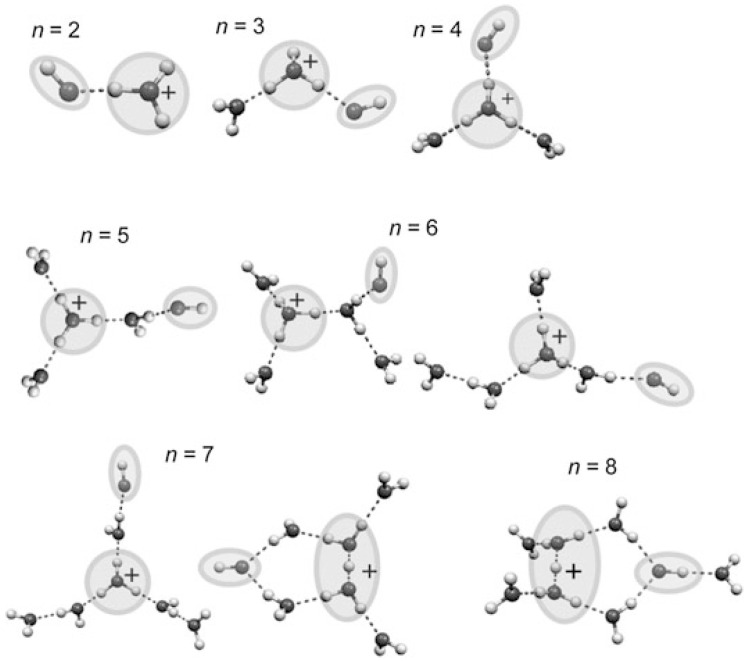
Experimentally characterized cluster structures of (H_2_O)_n_^+•^. Figure adapted from [50] with permission. Copyright 2013 Springer Nature.

As discussed above, the •OH radical water cluster cations (H_2_O)_n_^+•^ (n ≥ 3) should be regarded as “protonated water clusters with an •OH radical”, (H_2_O)_n−1_H^+^(•OH), as has been reported for (H_2_O)_2_^+•^ [99]. Mizuse et al. further reported the •OH radical in (H_2_O)_n_^+•^ to be separated from the protonated site by one water molecule in the n ≥ 5 clusters and attributed this separation to the •OH radical being a weaker hydrogen bond acceptor than is a water molecule, with the consequence being that the first solvation shell of the protonated site is preferentially filled with water [110]. Figure 29 presents experimentally-characterized structures of (H_2_O)_n_^+•^. As mentioned above, these clusters are easily constructed from the (H_2_O)_n_H^+^ structures by substituting one of the water molecules, which is the next neighbor of the charged site for •OH radical [110]. The reactivity of water exposed to ionizing radiation would be expected to depend on hydrogen bond network structures around the created radicals.

Note the increasing similarity of the (H_2_O)_n_^+•^ and (H_2_O)_n_H^+^ spectra with increasing size of the cluster, a feature that can be attributed the decrease in the relative weight of the •OH radical and the “bridging” water site. In this regard, it is particularly difficult to find any marked difference between the spectra of (H_2_O)_11_^+•^ and (H_2_O)_11_H^+^. However, the clear appearance of the marker band (as shown in Figure 28) and the clearly observed smooth changes between spectra with increasing n for n ≤ 10 strongly suggest that the trends continue in clusters larger than n = 10.

### 3.3. (H_2_O)_n_^+•^ Isomers in a Global Potential Energy Surface

In addition to the simulations of the observed structures discussed above in detail, it was also valuable to characterize other stable structures on the potential energy surface. Mizuse et al. carried out systematic explorations of stable isomers of (H_2_O)_3–8_^+•^ on the HF/6-31G(d) and B3LYP/6-31+G(d) potential energy surfaces by using the Global Reaction Route Map(GRRM) program [111,112,113,114,115,116]. They reported the B3LYP/6-31+G(d) calculations yielding 3, 15 and 82 stable isomers for (H_2_O)_3_^+•^, (H_2_O)_4_^+•^and (H_2_O)_5_^+•^, respectively. Re-optimization at the MPW1K/6-311++G(3df, 2p) level yielded isomers at each size with global minimum energies. Calculations at the HF/6-31G(d) level yielded 2, 9, 40, 62, 170 and 224 isomers for (H_2_O)_3–8_^+•^, respectively. The theoretical calculations indicated the structures with the H_3_O^+^ cation separated from the •OH radical to be lower in energy by 3–10 kJ/mol than those with H_3_O^+^ in contact with ^•^OH, even when adopting the same network shapes (i.e., chains and rings). This result suggests stabilization afforded by separating an ion from a radical to be a general phenomenon.

## 4. Chemical Properties of (H_2_O)_n_^+•^: Simulations and Experimental Studies

### 4.1. Proton Transfer to Form Hydroxyl Radicals

Ionization of water clusters (liquid, molecular beam or vapor) has been investigated using several techniques, and the reaction was indicated according to the results to follow the equations

(H_2_O)_n_ + I_p_ → [(H_2_O)_n_^+•^]_ver_ + e^−^ (Ionization) (6)

[(H_2_O)_n_^+•^]_ver_ → (H_3_O^+^)(•OH)(H_2_O)_n−2_ (Complex Formation)

→ H^+^(H_2_O)_n−1_ + •OH (•OH Dissociation) (7)

**Figure 30:**
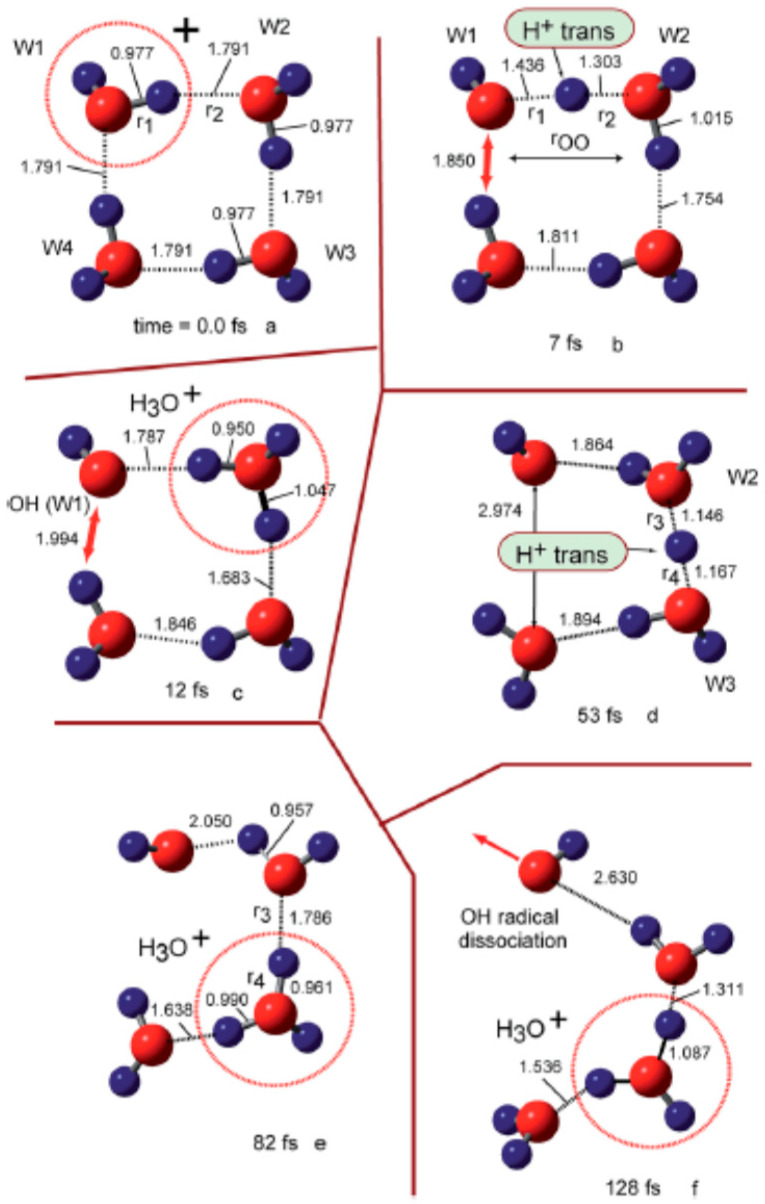
Snapshots of water tetramer cation (H_2_O)_4_^+•^ after vertical ionization from optimized structure. The values are bond distance in Å. Figure adapted from [118] with permission. Copyright 2015 The Royal Society of Chemistry.

**Figure 31:**
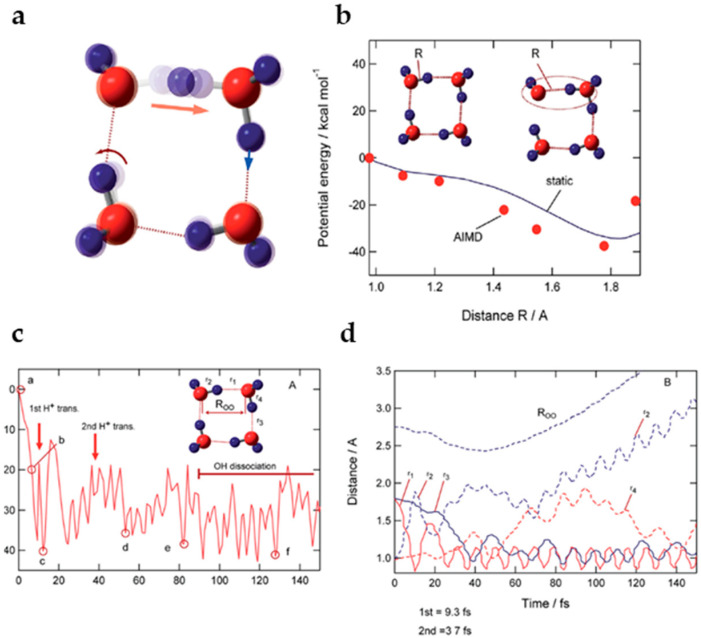
(**a**) Superposition of snapshots for the first proton transfer process. (**b**) Potential energy curve for the proton transfer process as derived from a static ab initio calculation (MP2/6-311++G (d, p) level). The values obtained from the direct *ab initio* molecular dynamics (AIMD) calculation are given as filled circles. (**c**) Potential energy and (**d**) geometrical parameters of the water tetramer cation following the vertical ionization each as a function of time. Figure adapted from [118] with permission. Copyright 2015 The Royal Society of Chemistry.

→ H^+^(H_2_O)_n−k_ + (k−1)H_2_O + •OH (•OH Dissociation + H_2_O Evaporation)

where I_p_ and ver denote the ionization potential and vertical ionized state from the parent neutral cluster, respectively. Based on a previous study in which the ionizations of water clusters (H_2_O)_n_ (n = 2–6) were investigated using the direct ab initio molecular dynamics (AIMD) method at the Hartree–Fock (HF) level [117], Tachikawa et al. performed the direct AIMD calculation on a more accurate potential energy surface (MP2/6-311++G(d,p) level) to estimate the rate of proton transfer along the hydrogen bond in the water cluster cation [118]. The rate of the first proton transfer was found to be strongly dependent on the cluster size: average time scales of proton transfer for n = 2, 3 and 4 were 28, 15 and 10 fs, respectively (from the MP2/6-311++G(d,p) level calculation), suggesting the proton transfer reactions to be very rapid processes in the three clusters. The clusters with n = 3 and 4 also each showed a second proton transfer (with average time scales of 120 fs and 40 fs, respectively, after the ionization), as shown in Figure 30 and Figure 31 for the case of the water tetramer (H_2_O)_4_^+•^.

As illustrated above, on the basis of the presented calculations, a reaction model of ionizations of the water clusters is proposed. Upon ionization of water, a hole would become localized on one of the water molecules of (H_2_O)_n_, and a proton would rapidly transfer—within a time scale of about 10 fs—from H_2_O^+•^ (W_1_^+•^) to H_2_O (W_2_) along the hydrogen bond. Next, the second proton transfer would occur from W_2_ (H^+^) to W_3_ (H_2_O) within a time scale of 50–100 fs. The second proton transfer would result in the separation of the •OH radical from H_3_O^+^ to form H_3_O^+^-H_2_O-^•^OH, as discussed in detail above in Section 3. Upon completion of this separation, any attraction between H_3_O^+^ and the •OH radical would be masked by the intervening H_2_O, immediately resulting in dissociation of the •OH radical from the system.

### 4.2. Initial Ultrarapid Charge Migration in the Chemistry of H_2_O^+•^

Generally, the •OH radical dissociated from H_2_O^+•^ is considered to be the main reactive species inducing one electron oxidation, •OH adduct formation and H atom abstraction reactions. In theory, H_2_O^+•^ should be considered as an ultra-oxidizing species. In the gas phase, oxidation of small molecules such as O_2_ has been reported, as discussed above [29,30]. In the condensed phase, a prerequisite for the direct oxidation process to be competitive is that the target molecule should be in close proximity to H_2_O^+•^; thus, the oxidation process induced by H_2_O^+•^ can be mimicked in highly concentrated solutions, where the nearest neighbors of H_2_O^+•^ may be molecules other than water [119,120]. By effectively solving the bottleneck, it was not possible to exclude the possibility of an •OH radical reaction in the oxidation process by using nanosecond or microsecond electron pulses as well as the problems of the absence of information on the yield of a direct effect of radiation on the solute, picosecond pulse radiolysis clearly showed that H_2_O^+•^ can also be considered as an ultra-oxidizing species in solution and presents a reactivity different from that of the •OH radical. Furthermore, several attempts have been made to experimentally investigate H_2_O^+•^ in different ways based on femtosecond laser technology [23,25,26].

**Figure 32:**
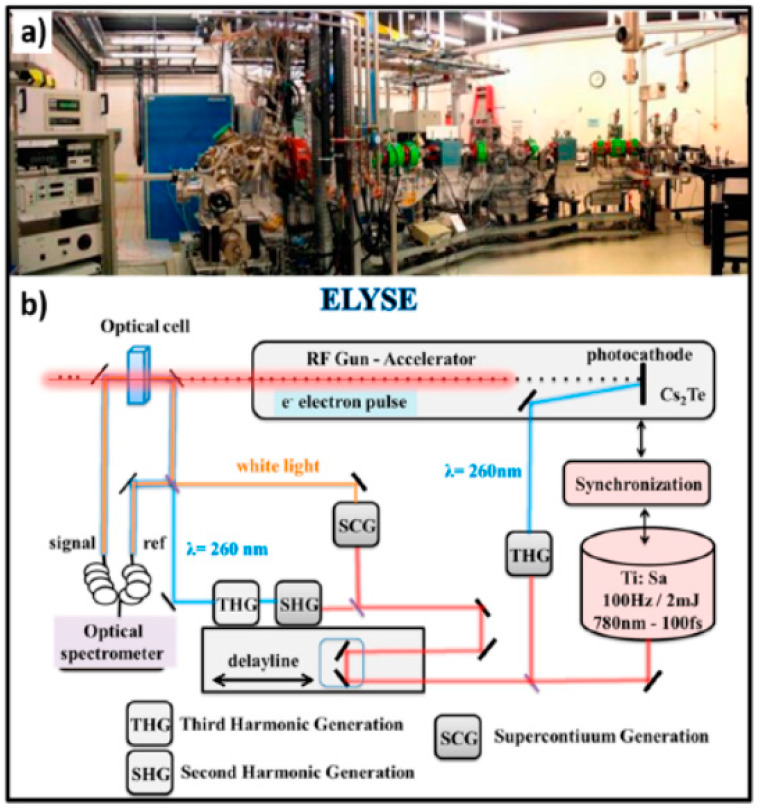
(**a**) Photograph of the pulse radiolysis facility (the only one in Europe) based on the ELYSE (a facility named after Lysis (Greek for degradation) by Electrons, which achieves a high energy (7–8 Mev) electron beam with a pulse width of 7 ps) picosecond pulsed electron accelerator from the Physical Chemistry Laboratory in Orsay, France. (**b**) Schematic description of the synchronization of the electron beam for ionization with a laser beam to probe the species created by the electron pulse. Figure adapted from [123] with permission. Copyright 2018 Multidisciplinary Digital Publishing Institute.

One of the femtosecond laser-driven accelerators established at ELYSE (a facility named after Lysis (Greek for degradation) by Electrons, which achieves a high energy (7–8 Mev) electron beam with a pulse width of 7 ps) is shown in Figure 32. This accelerator can achieve a high-energy (7–8 MeV) electron beam with a pulse width of 7 ps [121]. Its detection system is based on transient absorption spectroscopy with probe light, with wavelengths ranging from 380 nm to 1500 nm [122]. Therefore, the unique time-resolved technique based on a high-energy electron pulse enabled various investigators to explore ultra-rapid chemical reactions of H_2_O^+•^ through the scavenging method in a variety of highly concentrated aqueous solutions, i.e., by observing the formation of secondary radicals such as NO_3_^•^, SO_4_^•−^, X_2_^•−^ (X = Cl, Br) or H_2_PO_4_^•^. By further using diffusion-kinetic simulations of the spur reaction induced by the incident electrons, the first semi-quantitative estimation of the H_2_O^+•^ radicals scavenging fractions for a wide range of solutes could be established.

**Figure 33:**
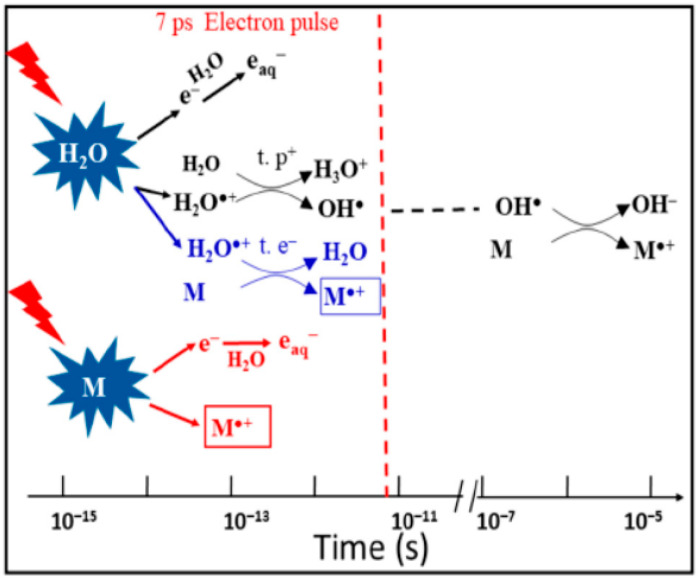
Schematic description of the reactions occurring in solutions containing a solute M at high concentration. Figure adapted from [123] with permission. Copyright 2018 Multidisciplinary Digital Publishing Institute.

H_2_O^+•^ has been suggested to be a stronger oxidative radical than other oxidants in aqueous solutions based on an estimation of its redox potential value: the standard redox potential of H_2_O^+•^/H_2_O couple has been recently estimated to be higher than 3 V vs. normal hydrogen electrode (NHE) [123]. A 7 ps pulse radiolysis set-up was used to demonstrate the oxidation reaction of H_2_O^+•^ in various solutions. As the lifetime of H_2_O^+•^ is too short for it to be observed directly, the product of the oxidation reaction was observed just after the electron pulse. The oxidation of M by H_2_O^+•^ and by direct effect gives M^+•^, which can be observed. Other oxidation reactions, such as oxidation by the •OH radical, do not occur so quickly. Therefore, the yield of M^+•^ measured precisely at 7 ps could provide information about its oxidation by H_2_O^+•^ (as shown in Figure 33).

Several studies in highly concentrated solutions were performed with the purpose of scavenging the radical cation of water, i.e., H_2_O^+•^ [124,125,126,127,128]. Highly concentrated sulfuric acid solutions were shown to be appropriate probe systems to elucidate the different mechanisms [129,130,131,132,133,134] and showed H_2_O^+•^ acting as a strong oxidant towards weak electron donors such as HSO_4_^−^ or H_2_PO_4_^−^ [135]. The reactivities of H_2_O^+•^ and D_2_O^+•^ were probed in hydrogenated and deuterated sulfuric acid solutions of various concentrations, combined with theoretical simulations by Wang et al. [136]

**Figure 34:**
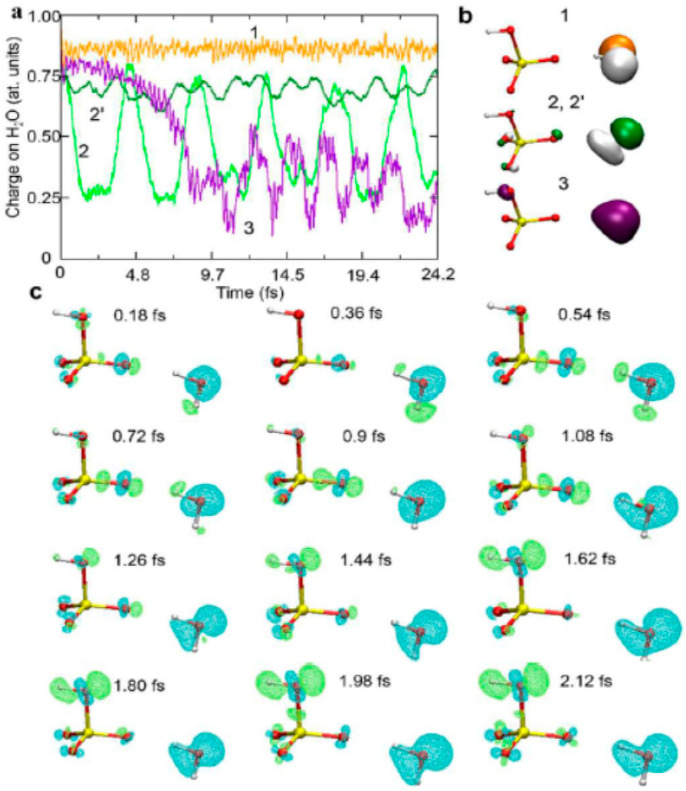
Electronic dynamics simulations. (**a**) Evolution of the charge of the water molecule hydrogen bonded to HSO_4_^−^ as a function of time after ionization took place, defined to be t = 0. Each plot (1, 2, 2′ and 3) corresponds to a different electronic dynamics generated by depopulating different valence MOs, the representations of which are shown in panel (**b**) for isosurfaces of ± 0.08 bohr^−3/2^. The two green curves (2 and 2′) correspond to ionization from the same MO but with different values of the hydrogen bond length between H_2_O and HSO_4_^−^, 1.8 and 2.4 Å, respectively, and (**c**) isosurfaces of the difference charge density with respect to the initial time for dynamics 2 and for different times spanning the first half period of charge migration. Blue and green isosurfaces indicate accumulation and depletion of electron density, respectively. Figure adapted from [136] with permission. Copyright 2017 The Royal Society of Chemistry.

**Figure 35:**
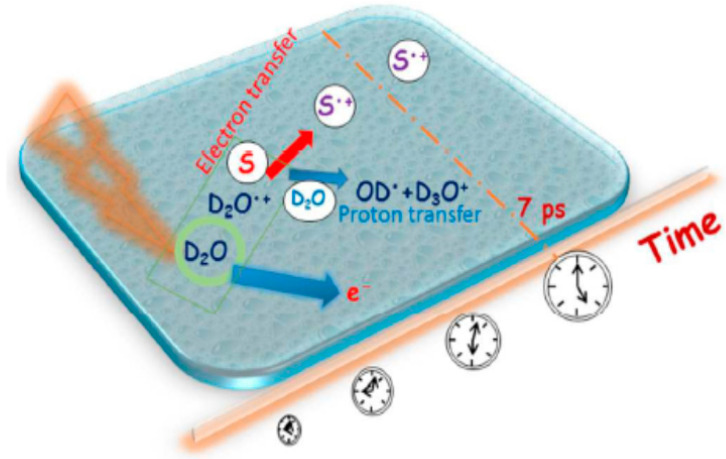
Schematic of the probing of the competition reactions of the radical cation D_2_O^+•^. A time resolution of 7 ps was used in the setup for probing the sulfate radical in deuterated sulfuric acid solutions. Figure adapted from [136] with permission. Copyright 2017 The Royal Society of Chemistry.

As illustrated in Figure 34 and Figure 35, electron transfer was indicated, based on the kinetics observed by Wang et al. [136], to be favored over proton transfer in competition reactions of the radical cation of water in deuterated solutions. These latest studies thus indicated the radical cation of water to be engaged not only in proton transfer reactions but also in redox reactions when H_2_O^+•^ (D_2_O^+•^) is formed in the vicinity of molecules different from water—with charge migration propelled by the excess energy present in the electron cloud just after ionization and not by nuclear motions as in standard chemical theories of electron transfer [136,137].

## 5. Summary and Outlook

### 5.1. Summary

1. The currently known methods and mechanisms of formation of (H_2_O)_n_^+•^ can be summarized as follows:
  - Method: wet nitrogen ionized as a result of being subjected to electron bombardment or corona discharge.
    Mechanism: a series of charge transfer reactions started by the formation of N_2_^+•^.
  - Method: photoionization of a water molecular beam or vapor, even including helium nanodroplets.
    Mechanism: (i) weakly bonded ions, e.g., (Ar)_k_(H_2_O)_n_^+•^, were cooled and then (H_2_O)_n_^+•^ were formed after further evaporating the carrier gas; (ii) the Franck–Condon factor could be enlarged due to the presence of carrier gas atoms in complex ions, (Ar)_k_(H_2_O)_n_^+•^, preventing the contraction of the O-O bond upon ionization. (H_2_O)_n_^+•^ were generated after the complex ions’ dissociation; (iii) rapid evaporation of the carrier gas could remove the excess energy in the (H_2_O)_n_^+•^; (iv) the excess energies remained in (H_2_O)_n_^+•^ after exposure to high-energy photos or particles would be removed by the exiting electron.
  - Method: photoionization by high-energy photons or 70 eV electrons impactions.
    Mechanism: the inner valence electrons could be excited and finally two H_2_O^+•^ ion radicals could be formed through an ICD process.
2. The current knowledge about the structure of (H_2_O)_n_^+•^ can be summarized as follows:
  - For (H_2_O)_2_^+•^, a theoretical simulation led to minimum-energy arrangements of both the structure resulting from proton transfer (H_3_O^+^-^•^OH) and the dimer cation structure [H_2_O…OH_2_]^+•^, but with the former indicated to be more stable than the latter by 8.8 kcal/mol with an interconversion barrier of 15.1 kcal/mol. Experimental results based on infrared spectroscopy and collision-induced dissociation MS spectroscopy also tended to support the formation of structures resulting from proton transfer.
  - A recently reported simulation of the dynamical process following water dimer ionization suggests that both the structure resulting from proton transfer and the dimer cation structures as well as other dissociated structures (H_3_O^+^ + •OH, H• + •OH + H_2_O^+•^) could be generated. The relative yields of these species are controlled by the populated electronic state of the radical cation. Proton transfer resulting from HOMO electron ionization is an ultrafast process, taking less than 100 fs; in the case of higher-energy ionization, the dynamical processes occur on longer timescales (200–300 fs).
  - For the larger (H_2_O)_n_^+•^ clusters (i.e., n ≥ 3), most of the simulations and experimental results indicated the presence of the structures resulting from proton transfer. At first, researchers expected •OH to be bonded tightly with H_3_O^+^. Later, however, they proposed that the •OH group would be separated from the protonated site by one water molecule since an •OH is a weaker hydrogen bond acceptor than is a water molecule. Then, researchers found a preference for the •OH to stay on the terminal site of the H-bond network—with, in the case of the clusters becoming bigger (n > 10), the •OH groups beginning to serve as H-bond donors (instead of acceptors) and in this way form H-bonds with water molecules.
3. It was recently found that, besides the ultra-fast proton transfer reaction between (H_2_O)_n_^+•^ and its neighboring water molecules to generate protonated water clusters and highly oxidative hydroxyl radicals, (H_2_O)_n_^+•^ should also be regarded as the strongest oxidant in the solution and that ultrafast charge migration could occur from a solute M to (H_2_O)_n_^+•^ in highly concentrated solutions. The reaction rate of charge migration was indicated by both the simulation and experimental results to be competitive with the rate of proton transfer.

### 5.2. Outlook

1. According to the electric field ionization theory, (H_2_O)_n_^+•^ may be expected to be produced under ambient conditions by the exposure of water vapor to the low-energy corona discharge in open air.
2. More accurate methods are needed to reliably predict the structures and chemical properties of (H_2_O)_n_^+•^.
3. Experimental investigation of chemical properties of (H_2_O)_n_^+•^ is greatly hindered due to the low amount of the generated (H_2_O)_n_^+•^ in current methods. New methods are needed to allow a higher abundance of (H_2_O)_n_^+•^.
4. Attosecond photoelectron spectroscopy based on high harmonic generation resolves H_2_O^+•^ decay in pure water and therefore shows high potential for the studies of (H_2_O)_n_^+•^.
5. Future studies of (H_2_O)_n_^+•^ reactivity need to take into account the competition of alternative reactions such as H_2_O^+•^ addition or H• abstraction with proton transfer and charge migration reactions.
6. More research on the reactivity of (H_2_O)_n_^+•^ in the condensed phase is essential in radio-biochemistry, nuclear industry and many other disciplines.
